# Long-term risks of major adverse cardiovascular events after acute kidney injury: a systematic review and meta-analysis

**DOI:** 10.1093/ckj/sfag145

**Published:** 2026-05-07

**Authors:** Denise M J Veltkamp, Finn Zwank, Wouter M Tiel Groenestege, Marianne C Verhaar, Wouter W van Solinge, Saskia Haitjema, Robin W M Vernooij, Roemer J Janse

**Affiliations:** Department of Nephrology and Hypertension, University Medical Center Utrecht, Utrecht University, Utrecht, The Netherlands; Central Diagnostic Laboratory, University Medical Center Utrecht, Utrecht University, Utrecht, The Netherlands; Central Diagnostic Laboratory, University Medical Center Utrecht, Utrecht University, Utrecht, The Netherlands; Central Diagnostic Laboratory, University Medical Center Utrecht, Utrecht University, Utrecht, The Netherlands; Department of Nephrology and Hypertension, University Medical Center Utrecht, Utrecht University, Utrecht, The Netherlands; Central Diagnostic Laboratory, University Medical Center Utrecht, Utrecht University, Utrecht, The Netherlands; Central Diagnostic Laboratory, University Medical Center Utrecht, Utrecht University, Utrecht, The Netherlands; Department of Nephrology and Hypertension, University Medical Center Utrecht, Utrecht University, Utrecht, The Netherlands; Central Diagnostic Laboratory, University Medical Center Utrecht, Utrecht University, Utrecht, The Netherlands; Julius Center for Health Sciences and Primary Care, University Medical Center Utrecht, Utrecht University, Utrecht, The Netherlands; Department of Nephrology and Hypertension, University Medical Center Utrecht, Utrecht University, Utrecht, The Netherlands; Central Diagnostic Laboratory, University Medical Center Utrecht, Utrecht University, Utrecht, The Netherlands

**Keywords:** acute tubular necrosis, cardiorenal syndrome, cardiovascular, chronic renal insufficiency, dialysis

## Abstract

**Background and aims:**

Acute kidney injury (AKI) is increasingly recognized as a long-term risk factor for chronic kidney disease and cardiovascular disease (CVD). However, it remains uncertain which patients with AKI are at greatest CVD risk. We performed a systematic review and meta-analysis to quantify CVD risks post-AKI and to determine whether these risks differ by AKI severity, duration, and clinical setting.

**Methods:**

PubMed and Embase were systematically searched for studies comparing individuals with and without AKI and reporting major adverse cardiovascular events (MACE) or individual outcomes such as myocardial infarction (MI), stroke, heart failure (HF), or cardiovascular mortality. Follow-up was at least 1 year. Relative risks (RRs) were pooled in meta-analyses using random-effect models. Subgroup and metaregression analyses were used to explore heterogeneity across patient and AKI characteristics, and clinical settings.

**Results:**

We included 54 studies comprising 1261 090 individuals, of whom 290 648 experienced AKI. Meta-analyses showed that AKI was associated with an RR of 1.97 [95% confidence interval (CI) 1.67–2.27] for MACE (13.9% overall incidence), 1.64 [95% CI 1.38–1.89] for MI (3.5% overall incidence), 1.36 [95% CI 1.13–1.59] for stroke (1.7% overall incidence), 1.92 [95% CI 1.67–2.16] for HF (3.5% overall incidence), and 1.86 [95% CI 1.59–2.13] for cardiovascular mortality (9.4% overall incidence), compared to patients without AKI. Elevated risks were observed across all AKI stages and durations and in patients across all studied clinical settings, including noncardiac care. The highest RRs were shown for more severe AKI stages and longer AKI durations. Older age and lower baseline estimated glomerular filtration rate were associated with even higher risk of MACE compared to patients without AKI.

**Conclusions:**

AKI is followed by an increase in CVD risk, even after AKI with low severity. These findings highlight AKI as a clinically relevant CVD risk marker and support the need for targeted post-AKI care management to prevent future cardiovascular events.

## Graphical Abstract

**Graphical Abstract:**
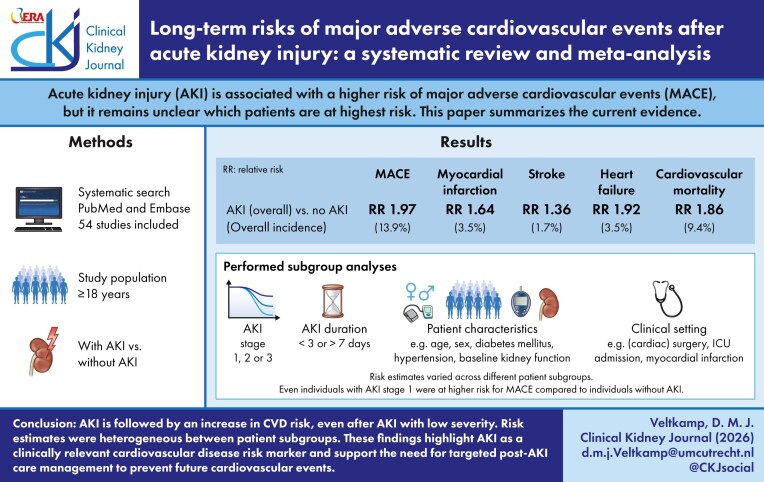

KEY LEARNING POINTS **What was known:**

- Acute kidney injury (AKI) is increasingly recognized as a long-term risk factor for chronic kidney disease (CKD) and, directly or indirectly through CKD, cardiovascular disease (CVD).

**This study adds:**

- The risks of CVD outcomes were higher among individuals with more severe AKI stages or longer AKI durations. Notably, even individuals with the lowest AKI stage or a short AKI duration had increased risks of these outcomes.
- Higher age and lower baseline estimated glomerular filtration rate were associated with increased risk of adverse cardiovascular outcomes post-AKI.
- Risks of a major adverse cardiovascular event (or a separate CVD outcome) after AKI were different between clinical settings with the highest relative risks in comparison to individuals without AKI in the context of cardiovascular surgery. Relative risks were also high in noncardiac care settings.

**Potential impact:**

- This review raises awareness of the association of AKI and CVD. Even individuals with the lowest AKI stage and short-lasting AKI showed increased risks.
- Risks for adverse cardiovascular outcomes were heterogeneous between patient subgroups, based on AKI stage, AKI duration, and clinical setting. This highlights the need to tailor post-AKI care strategies to prevent future cardiovascular events.

## INTRODUCTION

An abrupt worsening of kidney function, acute kidney injury (AKI), occurs in approximately 10%–20% of hospitalized individuals [1, 2]. AKI has a highly heterogeneous etiology and often occurs as a consequence of underlying disease or medical intervention. It is associated with increased risk of chronic kidney disease (CKD) incidence or progression [3–5]. Previous research has shown that CKD is strongly associated with increased risk of cardiovascular disease (CVD) [6]. However, the prognostic association of AKI with long-term CVDs, regardless the interference of CKD, remains less well established. Insights in long-term CVD risks post-AKI may contribute to the improvement of tailored care management protocols and facilitate shared decision-making for patients with AKI. Previous reviews on the association of AKI with cardiovascular outcomes either included a very limited amount of studies, combined distinct CVD outcomes into one outcome, focused on AKI in patients who underwent coronary angiography only, or included studies that defined AKI following a variety of gradings, other than the KDIGO (Kidney Disease: Improving Global Outcomes), AKIN (Acute Kidney Injury Network), or RIFLE (Risk, Injury, Failure, Loss, End-stage kidney disease, introduced by the Acute Dialysis Quality Initiative) criteria [7–10]. Importantly, since the last comprehensive review that reported on separate CVD outcomes, published in 2017 [9], more studies have been published that further specify the CVD risks based on certain patient and disease characteristics, such as AKI stage and duration, and in a wider variety of clinical settings. Thus, an update including the most recent evidence is appropriate.

In this systematic review, we analysed the association of AKI with major adverse cardiovascular events (MACE) in a large number of (newly published) studies using established AKI gradings and in various clinical settings. Also, the individual MACE outcomes were studied, including myocardial infarction (MI), heart failure (HF), stroke, and cardiovascular mortality. The results were stratified by AKI stage, AKI duration, and clinical setting. We additionally assessed effect modification by age, sex, baseline kidney function, diabetes mellitus, and hypertension on the association between AKI and cardiovascular outcomes.

## MATERIALS AND METHODS

### Protocol and search strategy

This systematic review and meta-analysis was registered in PROSPERO (CRD42023425871) and reported according to the Preferred Reporting Items for Systematic Reviews and Meta-analyses (PRISMA) guideline and Meta-analysis Of Observational Studies in Epidemiology guidelines [11–13]. We performed a search in PubMed and Embase, last updated on 25 September 2025. The search strategy included, among others, the terms AKI, MACE, MI, HF, stroke, and cardiovascular mortality (Supplementary Table S1).

### Exposure and outcomes

The exposure of interest in the study was AKI, and its definition was in accordance with the criteria established by KDIGO, AKIN, or RIFLE, as reported by the original studies [14–16]. RIFLE stages Risk, Injury, and Failure were analysed as KDIGO and AKIN stages 1, 2, and 3, respectively. The outcomes of interest were HF, MI, stroke, cardiovascular mortality, and MACE. MACE is a composite endpoint of all-cause and cardiovascular death and cardiovascular endpoints. Studies that reported a composite of CVD outcomes, but not naming it MACE, were also included as MACE (e.g. “major adverse cardiovascular and cerebrovascular events” or “any CVD outcome”).

### Screening and data extraction

Title and abstract screening as well as full text assessment were performed by F.Z., R.J.J., and D.M.J.V. Disagreements were resolved by discussion. Studies were considered eligible if (i) the study was an observational cohort study, (ii) AKI was defined using the KDIGO, AKIN, or RIFLE criteria, (iii) at least one of the outcomes of interest was reported, (iv) the minimum age was 18 years, (v) a control group was included containing individuals without AKI, and (vi) the follow-up time was at least 1 year post-AKI. Studies were excluded if (i) they were case-control studies, clinical trials (to prevent the influence of intervention from affecting our analyses), case reports, commentaries, study protocols, or conference abstracts, (ii) the clinical setting in which the AKI occurred was restricted to pregnancy, chronic liver disease, chronic HF, transplantation (solid organs or stem cells), nephrectomy, or COVID-19, (iii) the study was written in non-English, or (iv) the manuscript could not be retrieved.

### Data extraction and risk of bias assessment

Data were extracted by F.Z. and D.M.J.V. The risk of bias (ROB) assessment was performed by F.Z., R.W.M.V., and R.J. using an adaptation of the Newcastle–Ottawa Scale [17]. Herewith, the selection of the AKI and non-AKI study groups (e.g. representativeness of the cohort with individuals in clinical practice instead of the inclusion of a selected cohort), and the ascertainment of the outcome (e.g. adequate follow-up length or percentage of individuals loss to follow-up) were assessed using a “star system.” The comparability of the groups and adjustment for confounding (e.g. the groups were equal concerning age) were not assessed as ROB, as we focused on the association between AKI and CVD outcomes, rather than the causal pathway. However, relevant study characteristics are reported in the table of study characteristics, allowing readers to assess these aspects. The risk of immortal time bias (ITB) was added to the ROB analysis as ITB can greatly influence on the study outcome. For instance, we cannot observe AKI in individuals who would hypothetically experience an episode of AKI but die before. They are then assigned to the non-AKI group. As a result, individuals who are more likely to die early, for instance due to a high cardiovascular risk, are assigned to the non-AKI group, while the AKI group contains individuals who are less likely to experience these outcomes. In conclusion, the ROB assessment focused on the representativeness of the cohort, the length of follow-up, and the percentage of loss to follow-up. ITB was considered within the ROB analysis instead of evaluating group comparability, although the characteristics of the study groups are reported for transparency. Any study could obtain maximum four, two, and three stars per category, respectively. Studies with a total of seven or more stars were considered as high-quality studies, with five or six stars as moderate-quality studies, and below five stars as low-quality studies. Publication bias was evaluated by visually examining funnel plot asymmetry and by applying Egger’s regression test [18].

**Table 1:** Study characteristics.

								AKI		No AKI	AKI/no AKI					
Authors	Country	Setting	AKI definition	ROB	FU, years***	Loss to FU, %	Outcomes	AKI, *n*	Stage 1/2/3/2 or 3/KRT, %	No AKI, *n*	Age, years	Male, %	BL kidney function^****^	BL CKD ≥3A, %	DM, %	HT, %
Tsagalis *et al*. (2009) [61]	Greece	Stroke	AKIN	Medium	10	–	MACE	575	80/10.8/9.2/20/–	1580	72/70	61/61	177(SCr)/ 88(SCr)	68/25	27/25	71/69
Anzai *et al*. (2010) [62]	Japan	STEMI with PCI	KDIGO	High	3,3	–	MACE	31	–/–/–/–/–	110	69/61	77/89	66/76	–/–	29/39	68/48
Choi *et al*. (2010) [33]	United States	HIV	AKIN	Low	5,7	–	CVD, HF	3060	80.2/–/–/19.8/10.9	14 265	46/44	99/98	–/–	13/6	11/7	25/19
James *et al*. (2011) [30]	Canada	CA/PCI/CABG	AKIN	Medium	1,6	–	HF, MI, Stroke	1420	77.4/–/–/22.6/–	13 362	68/63	70/72	65/75	46/22	35/25	73/65
Kimura *et al*. (2011) [63]	Japan	CA/PCI	AKIN	Low	3,8	–	MACE**	81	–/–/–/–/–	2112	70/66	72/67	62/72	49/26	47/31	85/79
Adalbert *et al*. (2013) [57]	Romania	Peripheral artery surgery	AKIN	Medium	1	–	MACE	21	–/–/–/–/–	145	62/64	71/72	70/76	24/6	62/33	67/74
Hansen *et al*. (2013) [64]	Denmark	Cardiac surgery	AKIN	Low	5	0.2	MI, Stroke	287	82.9/–/–/17.1/–	743	70/64	73/73	94(SCr)/ 81(SCr)	–/–	20/14	58/56
Holzmann *et al*. (2013) [65]	Sweden	CABG	AKIN	Low	4,1	0	Stroke	2764	50.6/34/15/49/0	20 820	70/66	81/79	73/78	30/16	27/22	64/55
Olsson *et al*. (2013) [66]	Sweden	CABG	AKIN	Low	4,1	0	HF	2779	51.4/33.4/15.3/48.6/–	21 239	70/66	80/79	69/78	–/–	31/22	69/56
Gammelager *et al*. (2014) [32]	Denmark	ICU*	KDIGO	Low	3	0	HF, MI, Stroke	4792	55.6/–/–/44.4/–	16 764	68/57	61/54	181(SCr)/ 74(SCr)	19/6	17/9	25/13
Rydén *et al*. (2014) [67]	Sweden	CABG	AKIN	Low	5	–	MI	3529	88.8/11.2/2.8/14.1/–	24 300	–/–	–/–	–/–	–/–	–/–	–/–
Hansen *et al*. (2015) [27]	Denmark	Cardiac surgery	KDIGO	Low	2,7	–	HF, MACE, MI, Stroke	1457	77.1/–/–/22.9/–	3285	73/67	71/70	–/–	35/10	22/14	–/–
Ko *et al*. (2015) [21]	Japan	Type A acute aortic dissection	KDIGO	Low	2,6	0	MACE*	165	53.9/13.9/32.1/46.1/–	210	66/67	60/46	61/64	–/–	5/8	81/81
Mitchell *et al*. (2015) [34]	United States	Contrast-enhanced CT	AKIN	Low	1	12.6	MI, Stroke	97	57.1/48.6/32.9/81.4/–	561	–/–	–/–	–/–	–/–	–/–	–/–
Monseu *et al*. (2015) [22]	France	Hospital	KDIGO	Medium	5,8	5.5	CM, HF, MACE, MI, Stroke**	411	80/13.9/6.1/20/–	960	69/64	65/55	64/78	–/–	100/100	–/–
Saratzis *et al*. (2015) [68]	United Kingdom	Valvular heart surgery	AKIN	High	5,2	–	CM, MACE, Stroke	188	–/–/–/–/0	880	72/71	92/92	67/74	25/16	18/12	70/66
Huber *et al*. (2016) [69]	United States	Major vascular surgery	KDIGO	Medium	7	–	CM	1801	–/–/25.1/–/–	1717	66/63	64/63	–/–	19/9	20/22	–/–
Ozrazgat-Baslanti *et al*. (2016) [49]	United States	Major surgery	KDIGO	Medium	10	–	CM	20 025	–/–/–/–/–	29497	58/53	56/48	–/–	16/3	19/14	46/39
Andreis *et al*. (2017) [70]	Italy	CA/PCI	KDIGO	Medium	1	–	CM, MACE	69	82.6/7.2/10.1/17.4/–	911	72/66	71/72	61/79	55/16	32/19	80/75
Parikh *et al*. (2017) [25]	United States	Cardiac surgery	AKIN	Low	3,8	–	MACE	348	28.2/–/–/5.7/–	619	73/74	73/67	64/68	40/32	42/35	84/77
Valle *et al*. (2017) [26]	United States	PCI	AKIN	Low	1	–	MACE, MI	39 850	85.8/–/–/14.2/–	413 625	77/75	52/58	62/66	–/–	45/33	86/83
Bansal *et al*. (2018) [31]	United States	Hospital*	KDIGO	Low	2	–	HF	150 434	83/11/6/17/–	150 434	65/65	98/98	69/69	35/34	37/32	70/68
Go *et al*. (2018) [50]	United States	Hospital	KDIGO	Low	1	6	HF, MI, Stroke	31 245	–/–/–/–/–	115696	69/69	50/49	–/–	38/33	36/34	74/72
Leistner *et al*. (2018) [71]	Germany	PCI	KDIGO	High	1	45.2	CM, HF, MACE, MI, Stroke**	125	–/–/–/–/–	333	84/84	50/57	49/48	–/–	32/30	83/86
Lentini *et al*. (2018) [58]	Italy	Abdominal aortic aneurysm	KDIGO	Medium	3,5	0	MACE**	23	69.6/21.7/4.3/26.1/–	28	74/72	22/14	75/75	–/–	–/–	–/–
Wu *et al*. (2018) [24]	Hong Kong	Valvular heart surgery	KDIGO	Medium	2,6	–	HF, MACE**	178	–/–/–/–/–	161	68/63	50/33	–/–	–/–	27/14	32/18
Chalikias *et al*. (2019) [72]	Greece	Acute MI	KDIGO	Medium	5,6	36	MACE	84	69/9.5/7.1/16.7/–	434	67/62	60/82	84/95	38/10	33/25	64/56
Armijo *et al*. (2020) [73]	Spain	Valvular heart surgery	AKIN	Medium	2	14.6	CM, MACE, MI, Stroke	106	79.2/8.5/12.3/20.8/–	615	73/73	77/71	42/44	81/74	38/30	66/7
Lysak *et al*. (2020) [48]	United States	Major surgery	KDIGO	Low	5	–	CM	7817	–/–/–/–/–	8357	74/73	54/48	–/–	19/6	22/21	59/61
Cho *et al*. (2021) [74]	South Korea	Valvular heart surgery	KDIGO	Medium	1	6.8	HF, MACE, MI, Stroke	284	–/–/–/–/–	906	66/61	54/52	88/95	–/–	22/15	48/44
Ikizler *et al*., Brar *et al*., and MacLaughlin *et al*. (2021) (ASSESS-AKI) [52–54]	United States and Canada	Hospital	KDIGO	Low	4,5	10.6	HF, Major atherosclerotic cardiovascular events	769	71.9/15.3/12.7/28.1/–	769	64/66	66/50	70/70	40/40	40/25	–/–
Mezhonov *et al*. (2021) [75]	Russia	MI	KDIGO	Low	1	–	CM	55	83.6/14.5/1.8/16.4/–	213	66/61	55/81	70/79	35/18	18/16	91/81
Niittyvuopio *et al*. (2021) [47]	Finland	ICU	KDIGO	Low	5	0	CM	838	–/–/–/–/20.3	1598	64/61	66/62	76(SCr)/73(SCr)	8/4	24/20	55/42
Yan *et al*. (2021) [55]	China	Elective noncardiac surgery	KDIGO	Low	5	–	MI, Stroke	509	–/–/–/–/–	6764	61/56	67/55	109(SCr)/ 72(SCr)	24/100	20/21	100/100
Korczak *et al*. (2022) [76]	Poland	Valvular heart surgery	KDIGO	Medium	1	–	CM, MACE**	29	–/–/–/–/–	47	–/–	–/–	–/–	–/–	–/–	–/–
Ng *et al*. (2022) [23]	Hong Kong	PCI	KDIGO	Medium	5	–	CM, MACE, MI, Stroke**	3642	–/–/–/–/–	27997	–/–	–/–	–/–	–/–	–/–	–/–
Zhang *et al*. (2022) [77]	China	Left atrial appendage closure	KDIGO	Medium	3	–	MACE	27	–/–/–/–/–	485	71/69	44/61	71/76	41/18	33/21	93/67
Andonovic *et al*. (2023) [29]	United Kingdom	ICU	KDIGO	Low	2,3	0.4	MACE, Myocardial injury, Stroke	1340	–/–/–/–/–	2654	59/54	60/52	84/96	–/–	19/11	–/–
Chen *et al*. (2023) [78]	China	PCI	KDIGO	Medium	1,1	2.7	MI, Stroke	107	95.3/4.7/0/4.7/–	3523	67/62	75/76	95(SCr)/80(SCr)	43/12	61/30	72/61
Horne *et al*. (2023) [51]	United Kingdom	Hospital	KDIGO	Medium	5	3.3	HF	433	58.9/24.5/16.6/41.1/1.2	433	70/70	57/51	70/70	29/29	22/22	–/–
Li *et al*. (2023) [79]	China	CA	KDIGO	Medium	5	–	CM	4289	–/–/–/–/–	44670	64/62	61/71	77/82	33/21	32/32	42/53
Lunyera *et al*. (2023) [80]	United States	PCI	KDIGO	Medium	1	–	MACE, MI, Stroke**	865	–/–/–/–/–	8557	67/63	60/68	56/77	58/27	42/29	74/70
Florens *et al*. (2024) [59]	France	Hospital (sensitivity analysis*)	KDIGO	Low	5	–	MACE**	530	–/–/–/–/–	2503	–/–	–/–	–/–	–/–	–/–	–/–
Lee *et al*. (2024) [56]	Singapore	Hospital	KDIGO	Low	4,2	–	MACE**	219	–/–/–/–/–	1465	62/56	57/58	58/84	60/23	100/100	–/–
Nakamura *et al*. (2024) [28]	Japan	PCI	AKIN	Medium	4,4	–	HF, MACE, MI, Stroke	82	–/–/–/–/–	795	74/69	73/77	53/67	–/–	50/34	72/65
Ozaki *et al*. (2024) [81]	Japan	PCI	KDIGO	High	3	1.9	MACE*	365	–/–/–/–/–	2855	–/–	–/–	–/–	84/37	–/–	–/–
Ruzzarin *et al*. (2024) [82]	Italy	MI	KDIGO	High	1	10.2	HF, MACE, Stroke**	102	–/–/–/–/–	359	89/88	42/51	–/–	57/23	26/20	80/78
Tajti *et al*. (2024) [83]	Germany	Chronic total occlusion PCI	KDIGO	Low	1	–	MACE*	312	–/–/–/–/–	2395	70/65	82/84	66/76	42/20	38/28	94/85
Heitmann *et al*. (2025) [84]	Iceland	CABG	KDIGO	Medium	9,6	0	MACE, MI, Stroke**	459	16.4/1.6/2.1/3.7/4.6	1828	–/–	–/–	–/–	62/30	–/–	–/–
Huepenbecker *et al*. (2025) [60]	United States	Abdominal surgery	RIFLE	Medium	1,6	–	Any cardiac condition, MI	51	–/–/–/–/–	327	64/60	–/–	88(SCr)/88(SCr)	–/–	18/11	67/36
Nagaraja *et al*. (2025) [85]	United States	PCI	AKIN	Medium	10	–	MACE, MI	856	–/–/–/–/10.2	8343	72/68	63/71	–/–	20/7	46/31	86/79
Suzuki *et al*. (2025) [86]	Japan	PCI	KDIGO	Medium	2	–	HF, MACE, MI, Stroke**	723	–/–/–/–/–	7193	73/69	75/78	88(SCr)/88(SCr)	36/18	44/37	80/72

*Individuals with the outcome of interest already present at baseline were explicitly excluded (e.g. patients with pre-existing HF were excluded from HF analyses, and those with a prior MI from MI analyses).

**Including MACE+.

***Maximum, mean or median follow-up duration is reported, based on what the original study reported.

^****^Baseline kidney function is reported as eGFR in ml/min/1.73 m^2^ if not stated otherwise. Serum creatinine is expressed in µmol/l.

–Means not reported.

BL, baseline; CA, coronary angiography; CM, cardiovascular mortality; DM, diabetes mellitus; KF, kidney failure; FU, follow-up; HT, hypertension; KRT, kidney replacement therapy; OHCA, out of hospital cardiac arrest; P, prospective; R, retrospective; SCr, serum creatinine.

### Statistical analysesrisk

Meta-analyses were performed for each outcome. Since the studies represent different populations, in different healthcare settings, clinical heterogeneity was expected, and a random-effect model was deemed more appropriate than the fixed-effect model. Studies that reported event rates were included in the analyses, so relative risks (RRs) could be calculated. Studies involving the same cohort were included only once. In subgroup meta-analyses, the outcomes were studied by AKI stage, AKI duration, and clinical setting. Heterogeneity was assessed with the I^2^ statistic. In sensitivity analyses, we repeated the analyses in studies with low ROB. Another sensitivity analysis was performed using a more restrictive MACE definition (“MACE+”), encompassing at least cardiovascular and cerebrovascular events and cardiovascular or all-cause death. Studies that incorporated rarely included outcomes in their MACE definition were excluded from this analysis in order to make the definition homogeneous (e.g. peripheral artery disease, major bleeding, cardiac surgery, pulmonary or systemic embolism, chronic dialysis, and aortic rupture) (Supplementary Table S3).

Metaregression analyses were performed to analyse effect modification on the association between AKI and the outcomes. The analysed variables included mean age, the percentage of male individuals, mean baseline estimated glomerular filtration rate (eGFR), the proportion of individuals having diabetes mellitus or hypertension, follow-up duration, and study size. Studies were included in the metaregression analysis if they had a low or medium ROB.

A *P*-value below .05 was considered significant. Analyses were performed using R version 4.3.2 [19, 20].

## RESULTS

**Figure 1:**
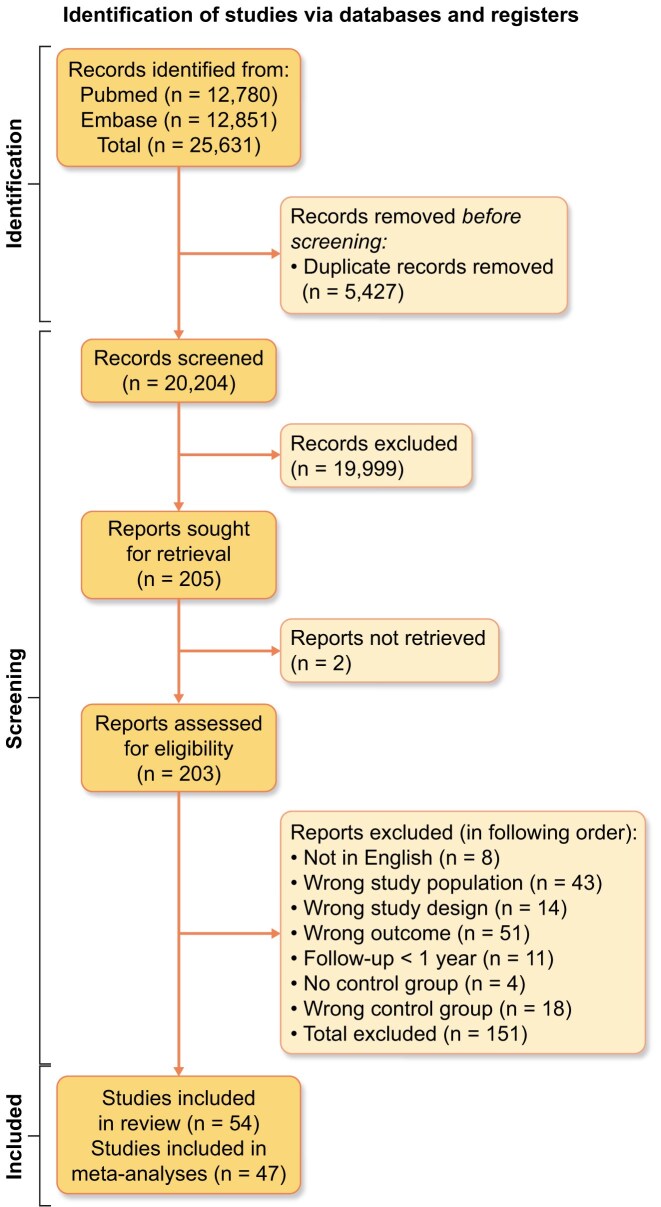
PRISMA flow diagram.

We identified 20 204 studies, of which 205 were assessed based on full text. In total, 54 studies were included in the review, which consisted of 52 different cohorts (Fig. 1). In total, 31 reported on MACE, 21 on MI, 21 on stroke, 16 on HF, and 13 on cardiovascular mortality. Reasons for exclusion in the full-text analysis are reported (Supplementary Table S2). Event rates were extractable from 47 studies, allowing meta-analyses to be performed.

Overall, 290 648 individuals with AKI and 970 442 individuals without AKI were included in the review (Table 1). The median sample size of the included studies was 422 [range 21–150 434] individuals with AKI, and 1658 [range 28–413 625] individuals without AKI (Supplementary Fig. S1). The mean reported mean/median age of the study participants was 68.8 [standard deviation (SD) 6.7] and 65.4 (SD 7.5) years, and the mean proportion of women across studies was 34.8% (SD 14.4) and 34.9% (SD 16.6) for individuals with and without AKI, respectively. In total, 23 studies were performed in Europe, 15 in Asia, and 14 in North America (Supplementary Fig. S2).

### Exposure and outcome definitions

Most studies defined AKI by the KDIGO criteria, 16 studies used the AKIN criteria, and 1 followed the RIFLE definition. The outcome definitions for the composite outcome MACE that were applied by all separate studies are reported in the Supplementary material (Supplementary Table S3). Definitions varied widely as most studies included cardiovascular and cerebrovascular events, but some studies reported “all-cause death” where other reported “cardiovascular death.” Some studies included HF in the composite outcome, and a minority of studies also incorporated other outcomes such as peripheral vascular disease or cardiovascular interventions.

### Risk of bias

ROB analysis showed that most studies were of moderate to high quality. Many studies did not report on loss to follow-up. ITB risk was high or not assessable in more than half of the studies (Supplementary Figs S4 and S5). Egger’s linear regression test and the funnel plots showed no significant funnel plot asymmetry, indicating that there was no evidence for publication bias (Supplementary Fig. S3).

### MACE

**Figure 2:**
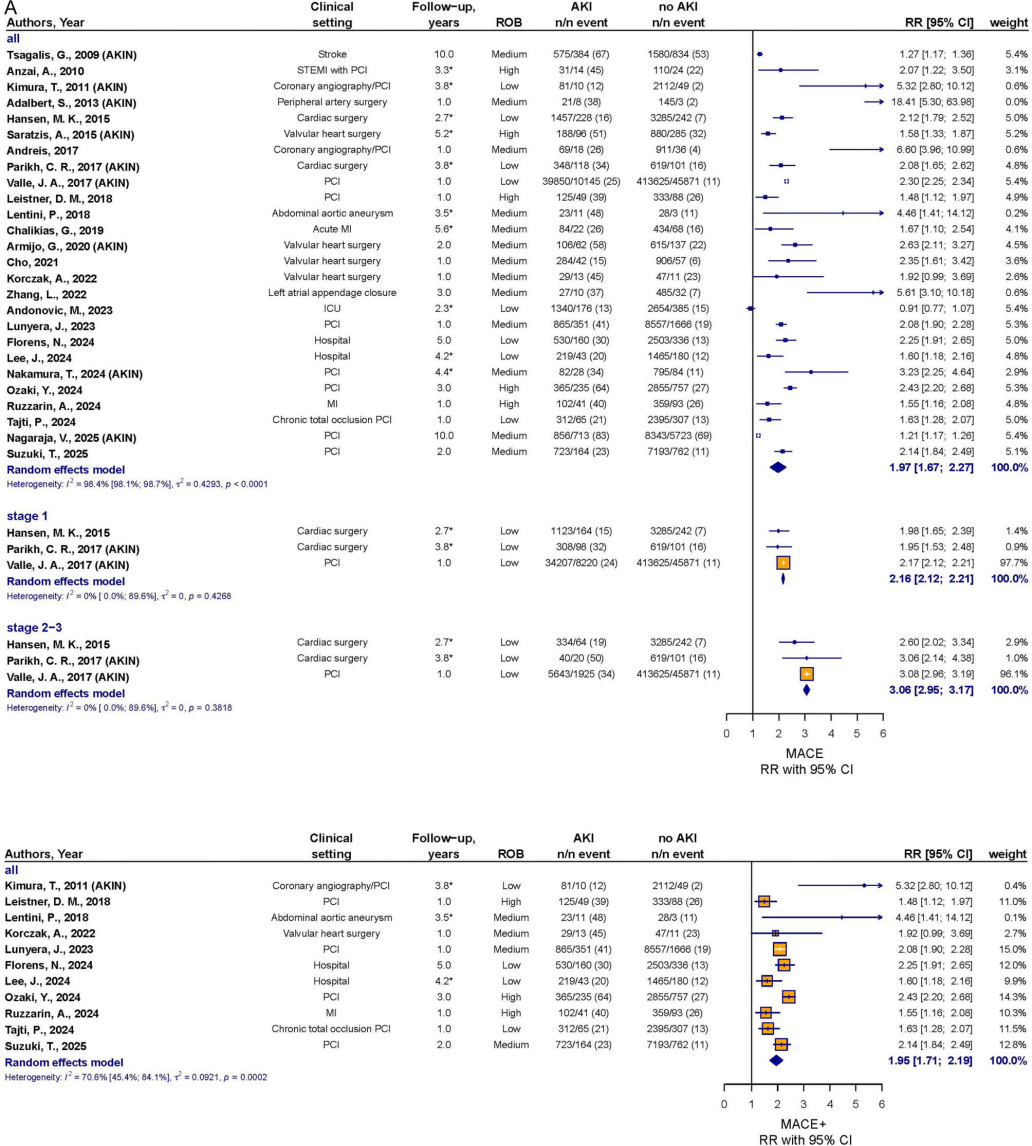
Forest plot on the association between AKI and MACE. Pooled outcomes are shown for all AKI stages together (all), and by AKI stage 1 or AKI stages 2 and 3. Results when only including studies with a low ROB: all stages RR 1.91 (95% CI 1.33–2.49); outcomes by AKI stage are the same as in this figure. Outcomes were not significantly different by AKI stages (*P* = .21). An asterisk (*) indicates studies in which the median or mean follow-up was reported, as no maximum follow-up duration was provided. CABG; coronary artery bypass grafting; MACE+, restrictive MACE definition, encompassing at least cardiovascular and cerebrovascular events and death. Studies that incorporated rarely included outcomes in their MACE definition were excluded from this analysis in order to make the definition homogeneous; STEMI, ST-elevation myocardial infarction.

In total, 31 studies reported on the composite outcome of MACE. Of these studies, 27 could be included in the meta-analysis, comprising 290 648 patients with AKI and 970 442 patients without AKI. Overall, MACE incidence was 27.0% in patients with AKI and 12.5% in patients without AKI. In total, four studies reported hazard ratios only [21–24]. As shown in the forest plots, the RR for MACE was 1.97 [95% confidence interval (CI) 1.67–2.27] in patients with AKI compared to patients without AKI. Results were comparable for MACE+. Of all studies, three stratified MACE risk by AKI stage. In this subset of studies, AKI stages 2 and 3 showed even an RR of 3.06 (95% CI 2.95–3.17) compared to no AKI (Fig. 2) [25–27]. Two studies reported separate event rates by AKI durations in the context of percutaneous coronary intervention (PCI) [28] and cardiac surgery [25]. They showed, compared to no AKI, a high RR for MACE in patients with persistent AKI [4.92 (95% CI 3.21–7.55)] [28] and in patients with longer AKI durations [7 and more days: 4.90 (95% CI 2.85–5.34)] [25]. Risks of MACE after AKI were different between clinical settings with the highest RRs in comparison to individuals without AKI in the context of cardiovascular surgery. Relative risks were also high in noncardiac care settings (Supplementary Table S4) [29].

### Heart failure

**Figure 3:**
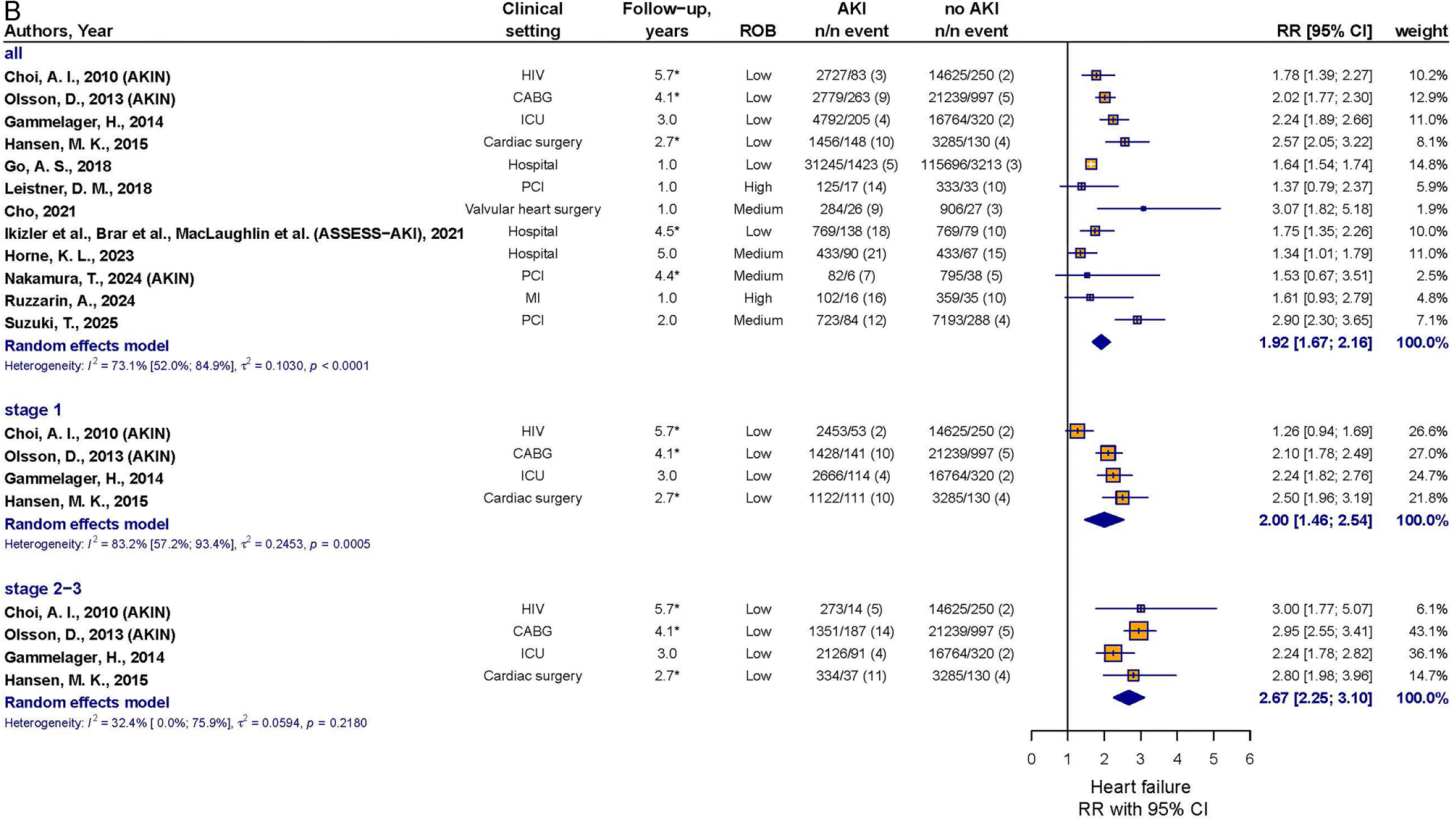
Forest plot on the association between AKI and HF. Pooled outcomes are shown for all AKI stages together (all), and by AKI stage 1 or AKI stages 2 and 3. Results when only including studies with a low ROB: all stages RR 1.95 (1.68–2.22); outcomes were not statistically different between AKI stages (*P* = .05). An asterisk (*) indicates studies in which the median or mean follow-up was reported, as no maximum follow-up duration was provided. CABG; coronary artery bypass grafting.

HF was analysed in 16 studies. In total, 12 studies comprising 45 851 patients with AKI and 182 037 patients without AKI, were included in the meta-analysis. Overall, HF incidence was 5.5% in patients with AKI and 3.0% in patients without AKI. In total, four studies reported no absolute event rates [22, 24, 30, 31]. In the meta-analysis, RR for HF was 1.92 (95% CI 1.67–2.16) in patients with AKI compared to patients without AKI. When risks were stratified by AKI stage, AKI stages 2 and 3 were associated with an RR of 2.67 (95% CI 2.25–3.10) (Fig. 3). Three studies reported on AKI duration and showed increased HF risk with increased AKI duration [28, 32, 33].

### Myocardial infarction

**Figure 4:**
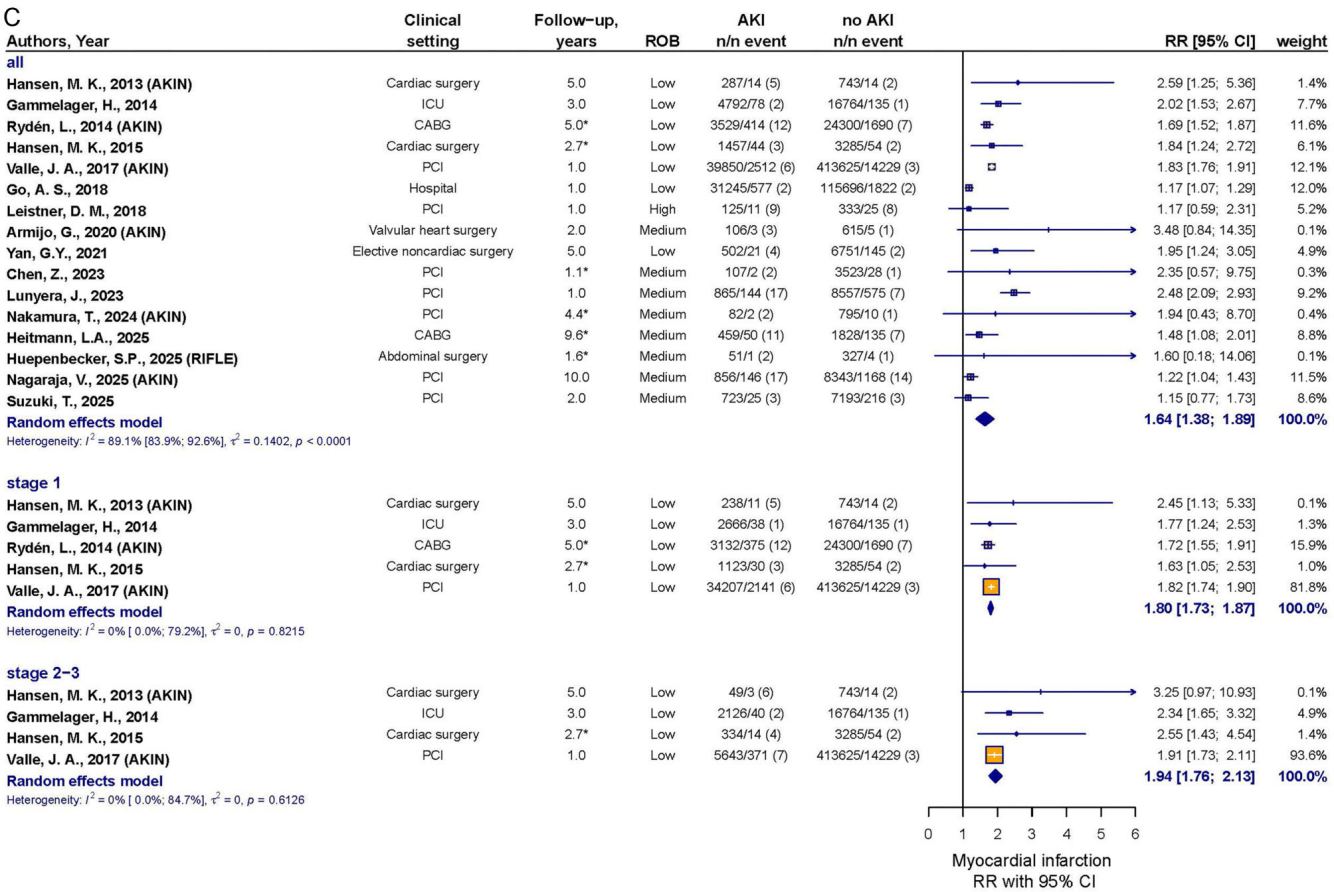
Forest plot on the association between AKI and MI. Pooled outcomes are shown for all AKI stages together (all), and by AKI stage 1 or AKI stages 2 and 3. Results when only including studies with a low ROB: all stages RR 1.72 (1.37–2.06); outcomes were not statistically different between AKI stages (*P* = .17). An asterisk (*) indicates studies in which the median or mean follow-up was reported, as no maximum follow-up duration was provided. CABG; coronary artery bypass grafting.

In total, 21 studies reported on MI of which 17 were eligible for meta-analysis, including 85 327 patients with AKI and 613 597 patients without AKI. Overall, MI incidence was 4.7% in the patients with AKI and 3.3% in patients without AKI. The other studies reported no event rates [22, 23, 30, 34]. Overall, the RR for MI was 1.64 (95% CI 1.38–1.89) in comparison to patients without AKI. The RR for MI in patients with AKI stages 2 and 3 was not significantly different than the increased risk in patients with AKI stage 1, compared to patients without AKI (*P*_interaction_ = .16) (Fig. 4). Longer AKI duration was associated with higher MI risk in intensive care unit (ICU) patients and patients who underwent PCI [28, 32]. The largest study showed that AKI without kidney function recovery before hospital discharge was associated with an RR of 2.75 (95% CI 1.73–4.37) for MI compared to no AKI [32].

### Stroke

**Figure 5:**
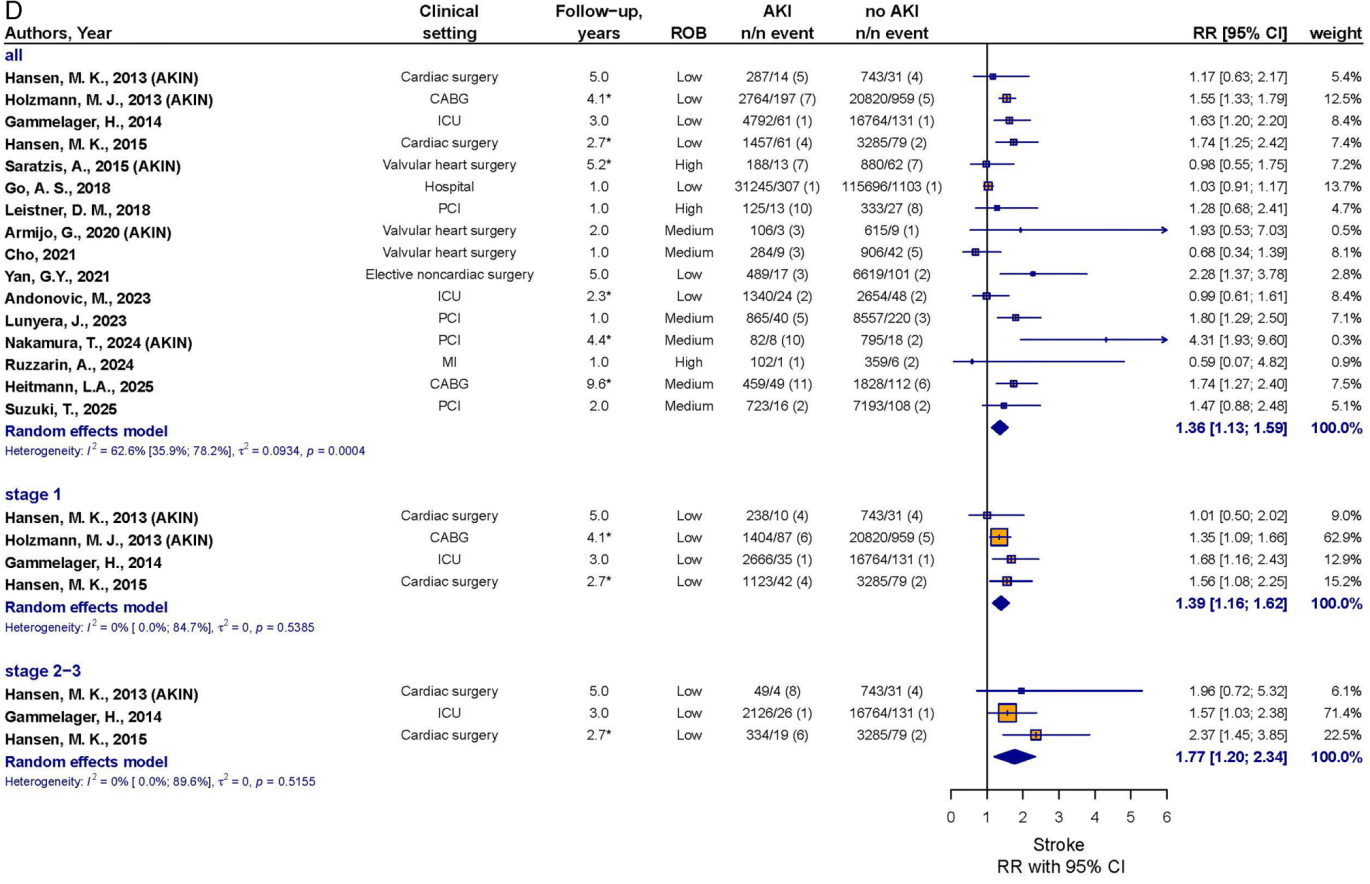
Forest plot on the association between AKI and stroke. Pooled outcomes are shown for all AKI stages together (all), and by AKI stage 1 or AKI stages 2 and 3. Results when only including studies with a low ROB: all stages RR 1.39 (1.08–1.69); outcomes were not statistically different between AKI stages (*P* = .46). An asterisk (*) indicates studies in which the median or mean follow-up was reported, as no maximum follow-up duration was provided. CABG; coronary artery bypass grafting.

Stroke was analysed in 21 studies and 17 studies were included in the meta-analysis. In total, 50 963 patients with AKI and 233 355 patients without AKI were included. Overall, stroke incidence was 2.0% in the patients with AKI and 1.7% in patients without AKI. Event rates could not be extracted from four studies [22, 23, 30, 34]. In total, the RR for stroke was 1.36 (95% CI 1.13–1.59) in patients with AKI compared to patients without AKI. Stroke risk increase was different for patients with AKI stage 1 than AKI stages 2 and 3, compared to patients without AKI (Fig. 5). Two studies reported on the association of AKI duration with stroke with conflicting outcomes [28, 32]. The largest study, which included ICU patients, showed no differences between patients with a recovered and nonrecovered kidney function at hospital discharge [32].

### Cardiovascular mortality

**Figure 6:**
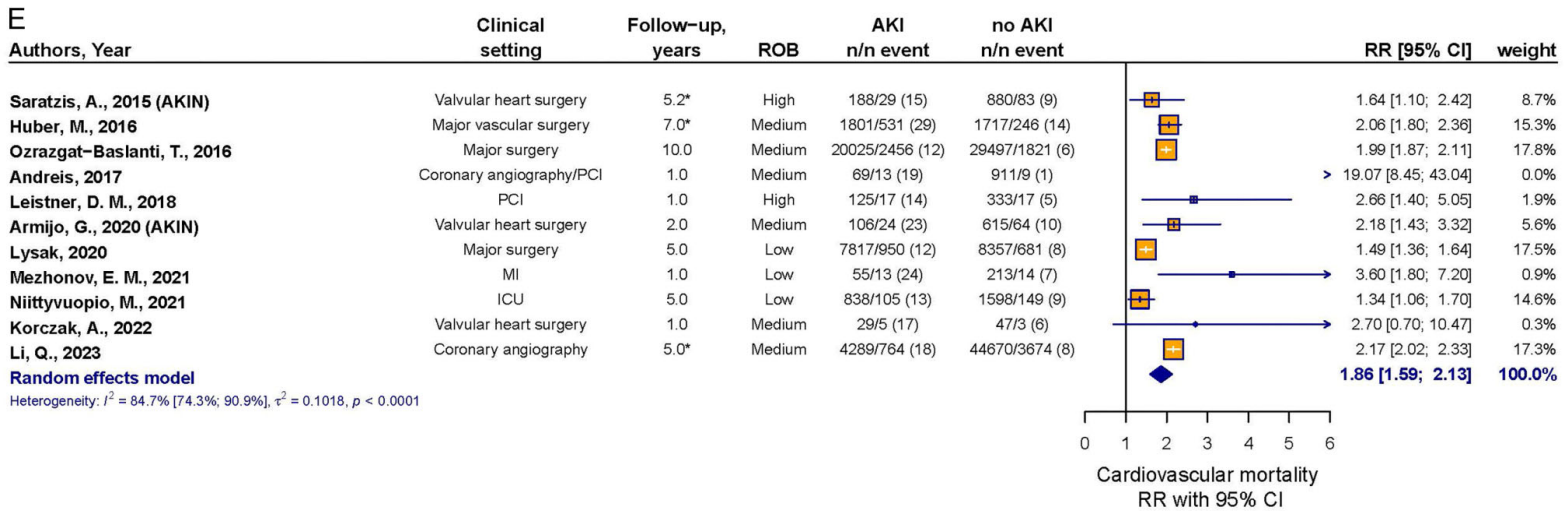
Forest plot on the association between AKI and cardiovascular mortality. Pooled outcomes are shown for all AKI stages together (all). No studies reported separate event rates by AKI stage. Results when only including studies with a low ROB: all stages RR 1.46 (1.22–1.69). An asterisk (*) indicates studies in which the median or mean follow-up was reported, as no maximum follow-up duration was provided. CABG; coronary artery bypass grafting.

Cardiovascular mortality was assessed in 13 studies, of which 11 were included in the meta-analysis, totaling 35 342 patients with AKI and 88 838 patients without AKI. Overall, cardiovascular mortality incidence was 13.9% in patients with AKI and 7.6% in patients without AKI. From two studies, the event rates were not reported [22, 23]. Overall, AKI was associated with an RR of 1.86 (95% CI 1.59–2.13) for cardiovascular mortality. No studies reported on the differences between AKI stages or durations (Fig. 6).

### Metaregression

**Figure 7:**
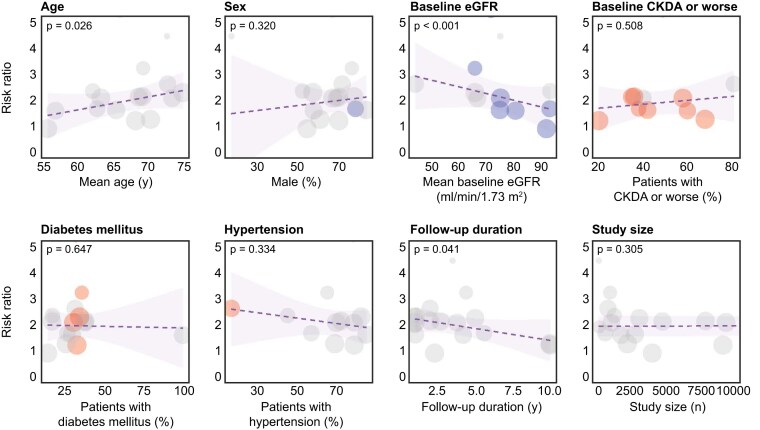
Metaregression lines to analyse the effect of covariates on the association between AKI and MACE. Every study is represented by a circle. Supplementary Fig. Size indicates the study’s weight in the random-effect model. The line indicates the regression line with 95% CIs. Studies were included in the metaregression analysis if they had a low or medium ROB. Blue and red indicate that the variable of interest was at least 10 percentage points lower or higher, respectively, in patients with AKI compared with patients without AKI (AKI as the reference group).

The metaregression analysis showed that higher age and lower eGFR at baseline were associated with higher RRs for MACE, thus being an effect modifier for the association between AKI and MACE. No effect modification was shown by sex, hypertension, or diabetes mellitus (Fig. 7). No strong effect modifiers were shown for the associations of AKI with the distinct cardiovascular outcomes (Supplementary Fig. S4).

### Sensitivity analysis

Outcomes in the meta-analyses including studies with low ROB differed in absolute numbers but conveyed the same clinical implications when only studies with a low ROB were included in the analyses (Figs 2–6).

## DISCUSSION

This review focused on long-term cardiovascular outcomes in patients with AKI compared to patients without AKI. AKI was associated with an RR of 1.97 for MACE. The increase in risks was consistent for individual cardiovascular outcomes, as we showed an RR of 1.92 for HF, 1.64 for MI, 1.36 for stroke, and 1.86 for cardiovascular mortality. Risk estimates were heterogeneous between patient subgroups, yet even individuals with AKI stage 1 had higher risks of MACE, HF, MI, and stroke, compared to individuals without AKI.

AKI has high incidence in clinical practice, especially AKI stage 1 and rapidly recovering AKI [1, 2]. Our findings indicate that AKI associated CVD outcomes may place a substantial burden on healthcare systems. Alarmingly, substantial underdiagnosis and undertreatment of AKI has been reported in clinical practice [35]. A recent study showed that only about half of the AKI survivors with CKD were prescribed guideline-recommended medications to prevent cardiorenal complications [36]. The underdiagnosis of AKI might improve by the implementation of an AKI alert. A recent systematic review reported that AKI documentation was increased after AKI-alert implementation, which might reflect better recognition [37–40]. Observational studies have shown that initiating preventive measures that may impede further cardiorenal consequences including blood pressure and glycemic control, use of renin–angiotensin–aldosterone system inhibitors (RAASi) and statins, and follow-up by nephrologist, are effective among AKI survivors [41–44]. So far, robust randomized clinical trials demonstrating improved cardiovascular outcomes post-AKI are lacking, although the initiation of RAASi is now being evaluated in an ongoing trial (NCT05272878).

The association between AKI duration and CVD outcomes could not be analysed in a meta-analysis, since the association was described in only a few studies and different definitions of kidney function recovery were used. Some studies categorize AKI duration using predefined thresholds (e.g. shorter or longer than 3 or 7 days), whereas others classify patients based on recovery status at hospital discharge. In addition, the definition of recovery is not consistent across studies. Some define recovery as a return to baseline kidney function, whereas others define recovery as kidney function within a certain percentage of the baseline value. Future studies applying standardized definitions of AKI duration and recovery are needed to better clarify how these features modify cardiovascular outcomes following AKI and to improve risk stratification in patients surviving an AKI episode. Nevertheless, longer AKI duration has been associated with higher rates of HF and (recurrent) MI among ICU patients [32], patients with HIV [33], patients with MI [45], and among all types of hospitalized patients in a 20-year Danish cohort study [46]. These findings collectively suggest associations between longer AKI duration and subsequent HF and MI. Notably, the studies that included a non-AKI cohort reported that patients with recovered kidney function post-AKI still exhibited increased risks compared with those without AKI, although mostly in combination with AKI stages 2 and 3 [32, 33, 45]. This further emphasizes the importance of early detection and appropriate clinical management of AKI.

Additionally, a minority of studies focused on the association of AKI with CVD in patients without cardiovascular comorbidities at baseline. Most studies in this review are performed in the context of cardiovascular interventions. A subgroup of the available studies was conducted in general hospital populations, in ICU settings, or in noncardiovascular surgery patients [22, 29, 31–34, 47–60]. AKI was associated with a higher risk of future CVD in these studies, including three that explicitly excluded individuals with pre-existing cardiovascular comorbidity at baseline [31, 32, 59]. These findings suggest that AKI identifies individuals at elevated CVD risk, even among those likely to have a lower baseline CVD risk than patients with prior CVD.

The MACE definitions used across studies were highly heterogeneous, which may influence reported effect sizes. Some studies included outcomes that are rarely incorporated in other MACE definitions, such as bleeding-related hospitalization, aortic rupture, dialysis initiation, pulmonary embolism, or myocardial injury. It is important to recognize which MACE definition was used when interpreting study results, as differences in definitions can substantially affect the reported outcomes. To facilitate transparency, we provided a detailed overview of the definitions used across studies. In this review, we addressed this issue by performing a sensitivity analysis including only studies with more similar MACE definitions, thereby reducing heterogeneity as much as possible. While complete harmonization was not feasible, this approach allowed for a more reliable assessment of the association between AKI and MACE. This sensitivity analysis did not show large differences compared with the results found in the main analysis. Additionally, we analysed the individual components of the composite outcome, offering further insight into the specific cardiovascular outcomes that are less affected by variability.

This review has its strengths and limitations. Stricter inclusion criteria regarding the definition of AKI and study outcomes, such as MACE, as previously described, could be applied compared to previous reviews since more studies were available [4, 9]. This improved the consistency and reliability of the pooled outcomes. Another strength of this review is the sensitive search strategy and inclusion of many studies allowing us to consider the heterogeneity of the results by performing subgroup analyses. We considered the risk of ITB for each study, which is a major concern when future information is used to select the study population or to assign individuals to the AKI or no-AKI group. However, some studies may also suffer from prevalent user bias (PUB), which we did not assess. PUB occurs when only patients who are still alive at the start of follow-up are included. In avoiding ITB, PUB can be induced (e.g. by analysing only individuals who survived until hospital discharge). By studying prevalent users, vulnerable patients who may have already had an outcome or have died are excluded. However, as most studies either started their follow-up at the moment of AKI detection and induced ITB, the presence of PUB was minor.

This review showed a wide heterogeneity in long-term CVD risks in individual with AKI. Future studies on CVD risks post-AKI might focus more on incorporating risks by patient profiles including various patient and AKI features, moving beyond single factor risks. This may improve the assessment of post-AKI CVD risk, as combinations of factors such as AKI severity, AKI duration, and comorbidities may provide a more nuanced risk stratification.

## CONCLUSION

AKI was associated with substantially elevated risks of major cardiovascular outcomes, including MACE, HF, MI, stroke, and cardiovascular mortality. Although risk levels varied across AKI stages, even patients with the mildest AKI remained at significantly increased risk for all outcomes examined. Higher age and lower baseline eGFR were associated with even more increased MACE risk. As the incidence of AKI is high, these findings underscore the need for individualized risk assessment and tailored post-AKI care strategies to improve cardiovascular outcomes in patients with AKI.

## Supplementary Material

sfag145_Supplemental_File

## Data Availability

All data that were used to support the findings of this study were obtained from previously published studies, and therefore, no data are deposited in a public repository. However, the extracted data used for the analyses are available upon request. For inquiries, please contact D.M.J.V. (d.m.j.veltkamp@umcutrecht.nl).
