# Supplementary material for: Long-term risks of major adverse cardiovascular events after acute kidney injury: a systematic review and meta-analysis
Source: Clin Kidney J. 2026 May 7;19(6):sfag145. doi: 10.1093/ckj/sfag145 (PMC13273423; doi:10.1093/ckj/sfag145)
Supplement: sfag145_Supplemental_File [file sfag145_supplemental_file.docx]

**Supplements**

**Supplemental Tables**

- **Table S1.** Search strategy
- **Table S2.** Reasons for exclusion in full-text assessment
- **Table S3.** MACE definitions by study
- **Table S4.** Meta-analysis outcomes, stratified by clinical setting

**Supplemental Figures**

- **Figure S1.** Sample size of included studies
- **Figure S2.** Countries of included studies
- **Figure S3.** Funnel plots
- **Figure S4.** Meta-regression plots
- **Figure S5.** Risk of bias assessment

**Supplemental Tables**

**Table S1.** Search strategy

**A. Search Medline through PubMed** *(September 25 2025)*

| **#** | **Query** |  |  |
| --- | --- | --- | --- |
| 1 | (“acute kidney injury”[Mesh] | OR |  |
|  | “acute kidney injur*”[ti] OR “acute on chronic kidney injur*”[ti] | OR |  |
|  | “acute kidney failure”[ti] OR “acute on chronic kidney failure”[ti] | OR |  |
|  | “acute kidney impairment”[ti] | OR |  |
|  | “acute kidney insufficienc*”[ti] | OR |  |
|  | “acute kidney disease”[ti] OR “acute on chronic kidney disease”[ti] | OR |  |
|  | “acute renal injur*”[ti] OR “acute on chronic renal injur*”[ti] | OR |  |
|  | “acute renal failure”[ti] OR “acute on chronic renal failure”[ti] | OR |  |
|  | “acute renal impairment”[ti] | OR |  |
|  | “acute renal insufficienc*”[ti] | OR |  |
|  | “acute renal disease*”[ti] OR “acute on chronic renal disease*”[ti] | OR |  |
|  | “acute tubular necros*”[ti] | OR |  |
|  | ((“surgery” [ti] OR “contrast” [ti] OR “sepsis” [ti] OR “IC”[ti] OR “intensive care” [ti]) AND (“kidney injur*”[ti] OR “renal injur*”[ti])) | OR |  |
|  | ((“surgery” [ti] OR “contrast” [ti] OR “sepsis” [ti] OR “IC”[ti] OR “intensive care” [ti]) AND (“kidney failure”[ti] OR “renal failure”[ti])) | OR |  |
|  | ((“surgery” [ti] OR “contrast” [ti] OR “sepsis” [ti] OR “IC”[ti] OR “intensive care” [ti]) AND (“kidney impairment”[ti] OR “renal impairment”[ti])) | OR |  |
|  | ((“surgery” [ti] OR “contrast” [ti] OR “sepsis” [ti] OR “IC”[ti] OR “intensive care” [ti]) AND (“kidney insufficienc*”[ti] OR “renal insufficienc*”[ti])) | OR |  |
|  | ((“surgery” [ti] OR “contrast” [ti] OR “sepsis” [ti] OR “IC”[ti] OR “intensive care” [ti]) AND (“kidney disease”[ti] OR “renal disease”[ti])) | OR |  |
|  | “AKIN“[ti] OR “RIFLE“[ti] OR (“KDIGO“[ti] AND “acute“[ti]) OR (“KDIGO“[ti] AND “AKI”[ti]) | OR |  |
|  | ”AKI”[ti] OR ”AKF”[ti] OR ”AKD”[ti] OR ”ARI” [ti] OR ”ARF”[ti] OR ”ARD”[ti] OR ”ATN”[ti] | ) |  |
|  | AND |  |  |
| 2 | ("Renal Insufficiency, Chronic"[Mesh] | OR |  |
|  | “chronic kidney disease”[tiab] | OR |  |
|  | “chronic kidney failure”[tiab] | OR |  |
|  | “chronic kidney impairment”[tiab] | OR |  |
|  | “chronic kidney insufficienc*”[tiab] | OR |  |
|  | “chronic renal disease”[tiab] | OR |  |
|  | “chronic renal failure”[tiab] | OR |  |
|  | “chronic renal impairment”[tiab] | OR |  |
|  | “chronic renal insufficienc*”[tiab] | OR |  |
|  | “end stage kidney disease”[tiab] OR “end stage renal disease”[tiab] OR “end stage renal failure”[tiab] | OR |  |
|  | “kidney failure”[tiab] OR “renal failure”[tiab] | OR |  |
|  | “CKD”[tiab] or “CKF”[tiab] or “CKI”[tiab] or “CRD”[tiab] or “CRF”[tiab] or “CRI”[tiab] or “ESRD”[tiab] or “ESRF”[tiab] or “ESKD”[tiab] or “ESKF”[tiab] | OR |  |
|  | "Cardiovascular Diseases"[Mesh] | OR |  |
|  | “cardiovascular disease*”[tiab] or “CVD”[tiab] or “heart failure”[tiab] or “myocardial infarc*”[tiab] or “MI”[tiab] or “stroke”[tiab] | OR |  |
|  | "Death"[Mesh] | OR |  |
|  | “death”[tiab] | OR |  |
|  | “mortality”[tiab] | OR |  |
|  | “survival”[tiab] | ) |  |
|  | AND |  |  |
| 3 | (“outcome*”[tiab] | OR |  |
|  | “progression”[tiab] | OR |  |
|  | “prognosis”[tiab] | OR |  |
|  | “follow up”[tiab] OR “followup”[tiab] or “follow-up”[tiab] | ) |  |
|  |  |  |  |
|  | #1 AND #2 AND #3 |  | 12.780 |

**B. Search Embase through Embase.com** *(September 25 2025)*

| **#** | **Query** |  |  |
| --- | --- | --- | --- |
| 1 | (‘acute kidney failure’/mj | OR |  |
|  | ‘acute kidney injury’:ti | OR |  |
|  | ‘acute kidney impairment’:ti | OR |  |
|  | ‘acute kidney insufficiency’:ti | OR |  |
|  | ‘acute kidney disease’:ti | OR |  |
|  | ‘acute kidney failure’:ti | OR |  |
|  | ‘acute renal injury’:ti | OR |  |
|  | ‘acute renal failure’:ti | OR |  |
|  | ‘acute renal impairment’:ti | OR |  |
|  | ‘acute renal insufficiency’:ti | OR |  |
|  | ‘acute renal disease’:ti | OR |  |
|  | ‘Acute tubular necrosis’:ti | OR |  |
|  | ‘acute-on-chronic kidney injury’:ti | OR |  |
|  | ‘acute on chronic kidney injury’:ti | OR |  |
|  | ((‘contrast‘:ti OR ‘surgery‘:ti OR ‘sepsis’:ti OR ‘IC’:ti OR ‘intensive care’:ti) AND (‘kidney injury‘:ti OR ‘renal injury’:ti)) | OR |  |
|  | ((‘contrast‘:ti OR ‘surgery‘:ti OR ‘sepsis’:ti OR ‘IC’:ti OR ‘intensive care’:ti) AND (‘kidney failure’:ti OR ‘renal failure’:ti)) | OR |  |
|  | ((‘contrast‘:ti OR ‘surgery‘:ti OR ‘sepsis’:ti OR ‘IC’:ti OR ‘intensive care’:ti) AND (‘kidney impairment’:ti OR ‘renal impairment’:ti)) | OR |  |
|  | ((‘contrast‘:ti OR ‘surgery‘:ti OR ‘sepsis’:ti OR ‘IC’:ti OR ‘intensive care’:ti) AND (‘kidney insufficiency’:ti OR ‘renal insufficiency’:ti)) | OR |  |
|  | ((‘contrast‘:ti OR ‘surgery‘:ti OR ‘sepsis’:ti OR ‘IC’:ti OR ‘intensive care’:ti) AND (‘kidney disease’:ti OR ‘renal disease’:ti)) | OR |  |
|  | ‘acute kidney tubular necrosis’:ti | OR |  |
|  | ‘AKI’:ti or ‘AKF’:ti or ‘AKD’:ti or ‘ARI’:ti or ‘ARF’:ti or ‘ARD’:ti or ‘ATN’:ti | ) |  |
|  | AND |  |  |
| 2 | ('chronic kidney failure'/exp | OR |  |
|  | 'chronic kidney impairment':ab,ti | OR |  |
|  | 'chronic kidney insufficiency':ab,ti | OR |  |
|  | ‘chronic renal disease':ab,ti | OR |  |
|  | 'chronic renal failure':ab,ti | OR |  |
|  | 'chronic renal impairment':ab,ti | OR |  |
|  | 'chronic renal insufficiency':ab,ti | OR |  |
|  | 'end stage renal disease'/exp | OR |  |
|  | ‘end stage renal failure':ab,ti | OR |  |
|  | 'end stage kidney disease':ab,ti OR 'end stage renal disease':ab,ti | OR |  |
|  | 'kidney failure':ab,ti | OR |  |
|  | 'ckd':ab,ti OR 'ckf':ab,ti OR 'cki':ab,ti OR 'crd':ab,ti OR 'crf':ab,ti OR 'cri':ab,ti OR 'esrd':ab,ti OR 'esrf':ab,ti OR 'eskd':ab,ti OR 'eskf':ab,ti | OR |  |
|  | 'cardiovascular disease'/exp | OR |  |
|  | 'cardiovascular disease':ab,ti or 'CVD':ab,ti or 'heart failure':ab,ti or 'heart infarction':ab,ti or 'myocardial infarction':ab,ti or 'MI':ab,ti or 'stroke':ab,ti | OR |  |
|  | 'death'/exp | OR |  |
|  | 'death':ab,ti | OR |  |
|  | 'mortality':ab,ti | OR |  |
|  | 'survival':ab,ti | ) |  |
|  | AND |  |  |
| 3 | (‘follow up’:ab,ti OR ‘followup’:ab,ti OR ‘follow-up’:ab,ti | OR |  |
|  | ‘progression’:ab,ti | OR |  |
|  | ‘outcome’:ab,ti | OR |  |
|  | ‘prognosis’:ab,ti | ) |  |
|  |  |  |  |
|  | #1 AND #2 AND #3 |  | 12.851 |

As this study is a sub study of a previous systematic review on long-term outcomes after AKI, the same search strategy was performed.^1^

**Table S2.** Reasons for exclusion in full-text assessment

| **Authors** | **Year** | **Reason for exclusion** |
| --- | --- | --- |
| Omotoso, B. A. et al. ^2^ | 2016 | PDF not found |
| Mezhonov, E. M. et al. ^3^ | 2018 | PDF not found |
| Bouzas-Mosquera, A. et al. ^4^ | 2007 | Not in English |
| Marrani, C. et al. ^5^ | 2012 | Not in English |
| Kobalava, Zh D. et al. ^6^ | 2015 | Not in English |
| Li, X. H. et al. ^7^ | 2017 | Not in English |
| Kremneva, L. V. and Suplotov, S. N. ^8^ | 2018 | Not in English |
| Kremneva, L. V. et al. ^9^ | 2022 | Not in English |
| Khruleva, Yu V. et al. ^10^ | 2023 | Not in English |
| Kremneva, L. V. et al. ^11^ | 2023 | Not in English |
| Welten, G. M. J. M. et al. ^12^ | 2007 | Wrong study population |
| Goldberg et al. ^13^ | 2010 | Wrong study population |
| Hedley, A. J. et al. ^14^ | 2010 | Wrong study population |
| Orii, K. et al. ^15^ | 2011 | Wrong study population |
| Maioli, M. et al. ^16^ | 2012 | Wrong study population |
| Ogita, M. et al. ^17^ | 2012 | Wrong study population |
| Ding, F. H. et al. ^18^ | 2013 | Wrong study population |
| Holzmann, M. J. et al. ^19^ | 2013 | Wrong study population |
| James, M. T. et al. ^20^ | 2013 | Wrong study population |
| Kume, K. et al. ^21^ | 2013 | Wrong study population |
| Currie, A. et al. ^22^ | 2014 | Wrong study population |
| Holzmann, M. et al. ^23^ | 2014 | Wrong study population |
| Watabe, H. et al. ^24^ | 2014 | Wrong study population |
| Wu, V. C. et al. ^25^ | 2014 | Wrong study population |
| Wu, V. C. et al. ^26^ | 2014 | Wrong study population |
| Arslan, S. et al. ^27^ | 2015 | Wrong study population |
| Chan, W. et al. ^28^ | 2015 | Wrong study population |
| Sato, A. et al. ^29^ | 2015 | Wrong study population |
| Attizzani, G. F. et al. ^30^ | 2016 | Wrong study population |
| Brown, J. R. et al. ^31^ | 2016 | Wrong study population |
| Park, S. D. et al. ^32^ | 2016 | Wrong study population |
| Tonkonogi, A. et al. ^33^ | 2016 | Wrong study population |
| Fortrie, G. et al. ^34^ | 2017 | Wrong study population |
| Nakada, Y. et al. ^35^ | 2017 | Wrong study population |
| Nakahashi, H. et al. ^36^ | 2017 | Wrong study population |
| Sato, A. et al. ^37^ | 2017 | Wrong study population |
| Shih, C. J. et al. ^38^ | 2017 | Wrong study population |
| Uzunhasan, I. et al. ^39^ | 2017 | Wrong study population |
| Ribitsch, W. et al. ^40^ | 2019 | Wrong study population |
| Pinier, C. et al. ^41^ | 2019 | Wrong study population |
| Adachi Y. et al. ^42^ | 2020 | Wrong study population |
| Peillex M. et al. ^43^ | 2021 | Wrong study population |
| Skalsky K. et al. ^44^ | 2021 | Wrong study population |
| Zhu, J. and Liu, W. ^45^ | 2021 | Wrong study population |
| Mohebi R. et al. ^46^ | 2022 | Wrong study population |
| Lu, J. Y. et al. ^47^ | 2023 | Wrong study population |
| Zhang, X.Y. et al. ^48^ | 2023 | Wrong study population |
| Presume J. et al. ^49^ | 2023 | Wrong study population |
| Marques da Silva, B. et al. ^50^ | 2024 | Wrong study population |
| Thanapongsatorn, P. et al. ^51^ | 2024 | Wrong study population |
| Tyl, B. et al. ^52^ | 2024 | Wrong study population |
| Khan, A.A. et al. ^53^ | 2025 | Wrong study population |
| Yang, T. Y. et al. ^54^ | 2025 | Wrong study population |
| Go, A. S. et al. ^55^ | 2010 | Wrong study design |
| Narula, A. et al. ^56^ | 2014 | Wrong study design |
| Tsai, H. S. et al. ^57^ | 2014 | Wrong study design |
| Crimi, G. et al. ^58^ | 2015 | Wrong study design |
| Giacoppo, D. et al. ^59^ | 2015 | Wrong study design |
| Usmiani, T. et al. ^60^ | 2016 | Wrong study design |
| Warren, J. et al. ^61^ | 2016 | Wrong study design |
| Geri, G. et al. ^62^ | 2018 | Wrong study design |
| Arbel Y. et al. ^63^ | 2019 | Wrong study design |
| Dieter B. P. et al. ^64^ | 2019 | Wrong study design |
| De Rosa, R. et al. ^65^ | 2021 | Wrong study design |
| Schytz P. A. et al. ^66^ | 2021 | Wrong study design |
| Kaneda K. et al. ^67^ | 2023 | Wrong study design |
| Landi, A. et al. ^68^ | 2023 | Wrong study design |
| Sesso, R. et al. ^69^ | 2004 | Wrong outcome |
| Parikh, C. R. et al. ^70^ | 2008 | Wrong outcome |
| Hobson, C. E. et al. ^71^ | 2009 | Wrong outcome |
| Brown, J. R. et al. ^72^ | 2010 | Wrong outcome |
| Ishibashi, Y. et al. ^73^ | 2010 | Wrong outcome |
| Kim, M. J. et al. ^74^ | 2011 | Wrong outcome |
| Wi, J. et al. ^75^ | 2011 | Wrong outcome |
| Brito, G. A. et al. ^76^ | 2012 | Wrong outcome |
| Bruetto, R. G. et al. ^77^ | 2012 | Wrong outcome |
| Zhou, Q. et al. ^78^ | 2012 | Wrong outcome |
| Choi, J. S. et al. ^79^ | 2013 | Wrong outcome |
| Hsieh, M. J. et al. ^80^ | 2013 | Wrong outcome |
| Lopez-Delgado, J. C. et al. ^81^ | 2013 | Wrong outcome |
| Neyra, J. A. et al. ^82^ | 2013 | Wrong outcome |
| Pannu, N. et al. ^83^ | 2013 | Wrong outcome |
| Ivert, T. et al. ^84^ | 2014 | Wrong outcome |
| Wonnacott, A. et al. ^85^ | 2014 | Wrong outcome |
| Abaci, O. et al. ^86^ | 2015 | Wrong outcome |
| Geri, G. et al. ^87^ | 2015 | Wrong outcome |
| Saratzis, A. et al. ^88^ | 2015 | Wrong outcome |
| Xu, J. R. et al. ^89^ | 2015 | Wrong outcome |
| Zhang, W. et al. ^90^ | 2015 | Wrong outcome |
| Centola et al. ^91^ | 2016 | Wrong outcome |
| Santana-Santos, E. et al. ^92^ | 2016 | Wrong outcome |
| Wang, X. et al. ^93^ | 2016 | Wrong outcome |
| Gupta, T. et al. ^94^ | 2017 | Wrong outcome |
| Kuji, S. et al. ^95^ | 2017 | Wrong outcome |
| Sawhney, S. et al. ^96^ | 2017 | Wrong outcome |
| Wu, B. et al. ^97^ | 2017 | Wrong outcome |
| Pourafkari et al. ^98^ | 2018 | Wrong outcome |
| Khatua, C. R. et al. ^99^ | 2019 | Wrong outcome |
| Kofman, N. et al. ^100^ | 2019 | Wrong outcome |
| Walther, C. P. et al. ^101^ | 2019 | Wrong outcome |
| Hertzberg, D. et al. ^102^ | 2020 | Wrong outcome |
| Holgado, J. L. et al. ^103^ | 2020 | Wrong outcome |
| Josa-Laorden, C. et al. ^104^ | 2020 | Wrong outcome |
| Khoury S. et al. ^105^ | 2020 | Wrong outcome |
| Lei L. et al. ^106^ | 2020 | Wrong outcome |
| Merdler, I. et al. ^107^ | 2021 | Wrong outcome |
| Crimi G. et al. ^108^ | 2022 | Wrong outcome |
| Fu, A. et al. ^109^ | 2022 | Wrong outcome |
| Huckaby L. V. et al. ^110^ | 2022 | Wrong outcome |
| Luo, X. et al. ^111^ | 2022 | Wrong outcome |
| Shemiesa, R. S. et al. ^112^ | 2022 | Wrong outcome |
| Tanaka T. et al. ^113^ | 2022 | Wrong outcome |
| Tanaka, T. et al. ^113^ | 2022 | Wrong outcome |
| Mutyala, S. C. V. S. et al. ^114^ | 2023 | Wrong outcome |
| Tarar, Z. I. et al. ^115^ | 2023 | Wrong outcome |
| Chen, Z. et al. ^116^ | 2024 | Wrong outcome |
| Choi, Una E. et al. ^117^ | 2024 | Wrong outcome |
| Kobalava, Zhanna D. et al. ^118^ | 2024 | Wrong outcome |
| Cioffi, G. et al. ^119^ | 2007 | Follow-up <1y |
| Szczech, L. A. et al. ^120^ | 2010 | Follow-up <1y |
| Schnabel, R. B. et al. ^121^ | 2015 | Follow-up <1y |
| Zhao, X. J. et al. ^122^ | 2017 | Follow-up <1y |
| Shiyovich A. et al. ^123^ | 2021 | Follow-up <1y |
| Wang, G. et al. ^124^ | 2021 | Follow-up <1y |
| Katsogridakis E. et al. ^125^ | 2022 | Follow-up <1y |
| Yandrapalli S. et al. ^126^ | 2022 | Follow-up <1y |
| Kutsal, D. A. and Terzi, S. ^127^ | 2024 | Follow-up <1y |
| Chou, C. L. et al. ^128^ | 2025 | Follow-up <1y |
| Mutlu, D. et al. ^129^ | 2025 | Follow-up <1y |
| Schiffl, H. et al. ^130^ | 2012 | No control group |
| Selby, N. M. et al. ^131^ | 2012 | No control group |
| Fernandes, A. R. et al. ^132^ | 2017 | No control group |
| Ohlmeier, J. et al. ^133^ | 2023 | No control group |
| Brar, S. et al. ^134^ | 2016 | Wrong control group |
| Omotoso, B. A. et al. ^2^ | 2016 | Wrong control group |
| Balogun, R. A. et al. ^135^ | 2017 | Wrong control group |
| Lee B. J. et al. ^136^ | 2018 | Wrong control group |
| Park S. et al. ^137^ | 2018 | Wrong control group |
| Rodríguez E. et al. ^138^ | 2018 | Wrong control group |
| Lee S. et al. ^139^ | 2019 | Wrong control group |
| Amatruda J. G. et al. ^140^ | 2021 | Wrong control group |
| Vasquez-Rios G. et al. ^141^ | 2021 | Wrong control group |
| Chen J. J. et al. ^142^ | 2022 | Wrong control group |
| Mansour S. G. et al. ^143^ | 2022 | Wrong control group |
| Cabrera Cárdenas, L. et al. ^144^ | 2023 | Wrong control group |
| Hirano, T. et al. ^145^ | 2024 | Wrong control group |
| Jensen, S. K. et al. ^146^ | 2024 | Wrong control group |
| Pan, H. C. et al. ^147^ | 2024 | Wrong control group |
| Wang, D. et al. ^148^ | 2024 | Wrong control group |
| Ahsan, S. I. et al. ^149^ | 2025 | Wrong control group |

**Table S3.** MACE definitions by study

| **Authors** | **Cardiovascular event** | **Cerebrovascular event** | **Death** | **HF** | **Coronary revascularization** | **Other** |
| --- | --- | --- | --- | --- | --- | --- |
| Adalbert, S. et al. ^150^ (2013) | x^a^ | x^a^ | - | - | - |  |
| Andonovic, M. et al. ^151^ (2023) | - | x^b^ | - | - | x | Myocardial injury |
| Andreis et al. ^152^ (2017) | x | x | C | x | - |  |
| Anzai, A. et al. ^153^ (2010) | x | - | C | x | x |  |
| Armijo, G. et al. ^154^ (2020) | x | x | A | - | - | Major bleeding; Cardiac surgery |
| Chalikias, G. et al. ^155^ (2019) | x | - | - | x | x | Pulmonary embolism |
| Cho et al. ^156^ (2021) | x | x | - | x | - |  |
| Florens, N. et al. ^157^ (2024) | x | x | C | x | - |  |
| Hansen, M. K. et al. ^158^ (2015) | x | x | - | x | - |  |
| Heitmann, L.A. et al. ^159^ (2025) | x | x | C | - | x |  |
| Kimura, T. et al. ^160^ (2011) | x | x | A | x | - |  |
| Ko, T. et al. ^161^ (2015) | x | x | A | - | - |  |
| Korczak, A. et al. ^162^ (2022) | - | x | C | - | - | Cardiovascular hospitalization |
| Lee, J. et al. ^163^ (2024) | x | x | C | - | - |  |
| Leistner, D. M. et al. ^164^ (2018) | x | x | A | x | - |  |
| Lentini, P. et al. ^165^ (2018) | x | x | C | x | x |  |
| Lunyera, J. et al. ^166^ (2023) | x | x | A | - | x |  |
| Monseu, M. et al. ^167^ (2015) | x | x | C | - | - |  |
| Nagaraja, V. et al. ^168^ (2025) | x | - | A | - | x |  |
| Nakamura, T. et al. ^169^ (2024) | x | x | A | x | - | Initiation of maintenance dialysis |
| Ng, A. K. Y. et al. ^170^ (2022) | x | x | A | - | x |  |
| Ozaki, Y. et al. ^171^ (2024) | x | x | A | x | - |  |
| Parikh, C. R. et al. ^172^ (2017) | x | - | A | x | x |  |
| Ruzzarin, A. et al. ^173^ (2024) | x | x | A | x | x |  |
| Saratzis, A. et al. ^174^ (2015) | x | x | C | x | - | Peripheral vascular events |
| Suzuki, T. et al. ^175^ (2025) | x | x | A | x | - |  |
| Tajti, P. et al. ^176^ (2024) | x | x | A | - | - |  |
| Tsagalis, G. et al. ^177^ (2009) | x | x | A | x | - | Thoracic or abdominal aortic rupture; Peripheral arteriopathy |
| Valle, J. A. et al. ^178^ (2017) | x | - | A | - | - | Bleeding-related hospitalization |
| Wu, M. Z. et al. ^179^ (2018) | x | x | A | x | - |  |
| Zhang, L. et al. ^180^ (2022) | - | x | C | x | - | Major bleeding; cardiac surgery |

A, all-cause death; C, Cardiovascular death; HF, Heart failure; MACE, major adverse cardiovascular event; MI, Myocardial infarction. A) non-fatal; b) Radiological evidence for stroke

**Table S4A.** Meta-analysis outcomes, stratified by clinical setting - clustering

|  | **Clinical setting clusters** | | | | | | |
| --- | --- | --- | --- | --- | --- | --- | --- |
| **Clinical setting** | **MI/PCI/CA** | **Cardiac surgery** | **Major vascular surgery** | **Other non-cardiac surgery** | **ICU** | **Hospital** | **Other** |
| MI |  |  |  |  |  |  |  |
| PCI |  |  |  |  |  |  |  |
| Acute MI |  |  |  |  |  |  |  |
| Chronic total occlusion PCI |  |  |  |  |  |  |  |
| Coronary angiography |  |  |  |  |  |  |  |
| Coronary angiography/PCI |  |  |  |  |  |  |  |
| STEMI with PCI |  |  |  |  |  |  |  |
| CABG |  |  |  |  |  |  |  |
| Cardiac surgery |  |  |  |  |  |  |  |
| Left atrial appendage closure |  |  |  |  |  |  |  |
| Valvular heart surgery |  |  |  |  |  |  |  |
| Abdominal aortic aneurysm |  |  |  |  |  |  |  |
| Major vascular surgery |  |  |  |  |  |  |  |
| Type A acute aortic dissection |  |  |  |  |  |  |  |
| Abdominal surgery |  |  |  |  |  |  |  |
| Elective noncardiac surgery |  |  |  |  |  |  |  |
| Major surgery |  |  |  |  |  |  |  |
| Peripheral artery surgery |  |  |  |  |  |  |  |
| ICU |  |  |  |  |  |  |  |
| Hospital |  |  |  |  |  |  |  |
| Contrast-enhanced CT |  |  |  |  |  |  |  |
| Coronary angiography/PCI/CABG |  |  |  |  |  |  |  |
| HIV |  |  |  |  |  |  |  |
| Stroke |  |  |  |  |  |  |  |

**Table S4B.** Meta-analysis outcomes, stratified by clinical setting - results

|  |  |  | **AKI** |  | **No AKI** |  |  | |
| --- | --- | --- | --- | --- | --- | --- | --- | --- |
| **Setting (clustered)** | **Studies (n)** | **FU in years, range** | **n** | **Outcome risk, range** | **n** | **Outcome risk, range** | **Pooled relative risk** | |
| **Major adverse cardiac event** | | | | | | | |  |
| MI/PCI/CA | 13 ^152^ ^153^ ^155^ ^160^ ^164^ ^166^ ^168^ ^169^ ^171^ ^173^ ^175^ ^176^ ^178^ | 1 – 10y | 43545 | 12.3 – 83.3% | 448022 | 2.3 – 68.6% | 2.18 (1.72-2.76), p < 0.01 | |
| Cardiac surgery | 7 ^154^ ^156^ ^158^ ^162^ ^172^ ^174^ ^180^ | 1 – 5.2y | 2439 | 14.8 – 58.5% | 6837 | 6.3 – 32.4% | 2.27 (1.8-2.86), p < 0.01 | |
| Major vascular surgery | 1 ^165^ | 3.5y | 23 | 47.8% | 28 | 10.7% | 4.46 (1.41-14.12), p = 0.01 | |
| Other non-cardiac surgery | 1 ^150^ | 1y | 21 | 38.1% | 145 | 2.1% | 18.41 (5.3-63.98), p < 0.01 | |
| ICU | - | - | - | - | - | - | - | |
| Hospital | 2 ^157^ ^163^ | 4.2 – 5y | 749 | 19.6 – 30.2% | 3968 | 12.3 – 13.4% | 1.94 (1.39-2.71), p < 0.01 | |
| **Myocardial infarction** |  |  |  |  |  |  |  | |
| MI/PCI/CA | 7 ^181^ ^164^ ^166^ ^168^ ^169^ ^175^ ^178^ | 1 – 10y | 42608 | 1.9 – 17.1% | 442369 | 0.8 – 14% | 1.6 (1.2-2.14), p < 0.01 | |
| Cardiac surgery | 5 ^154^ ^182^ ^158^ ^159^ ^183^ | 2 – 9.6y | 5838 | 2.8 – 11.7% | 30771 | 0.8 – 7.4% | 1.69 (1.54-1.86), p < 0.01 | |
| Major vascular surgery | - | - | - | - | - | - | - | |
| Other non-cardiac surgery | 2 ^184^ ^185^ | 1.6 – 5y | 553 | 2 – 4.2% | 7078 | 1.2 – 2.1% | 1.93 (1.25-3), p < 0.01 | |
| ICU | 1 ^186^ | 3y | 4792 | 1.6% | 16764 | 0.8% | 2.02 (1.53-2.67), p < 0.01 | |
| Hospital | 1 ^187^ | 1y | 31245 | 1.8% | 115696 | 1.6% | 1.17 (1.07-1.29), p < 0.01 | |
| **Stroke** |  |  |  |  |  |  |  | |
| MI/PCI/CA | 5 ^164^ ^166^ ^169^ ^173^ ^175^ | 1 – 4.4y | 1897 | 1 – 10.4% | 17237 | 1.5 – 8.1% | 1.76 (1.24-2.49), p < 0.01 | |
| Cardiac surgery | 7 ^154^ ^156^ ^182^ ^158^ ^159^ ^19^ ^174^ | 1 – 9.6y | 5545 | 2.8 – 10.7% | 29077 | 1.5 – 7% | 1.52 (1.35-1.71), p < 0.01 | |
| Major vascular surgery | - | - | - | - | - | - | - | |
| Other non-cardiac surgery | 1 ^185^ | 5y | 489 | 3.5% | 6619 | 1.5% | 2.28 (1.37-3.78), p < 0.01 | |
| ICU | 2 ^151^ ^186^ | 2.3 – 3y | 6132 | 1.3 – 1.8% | 19418 | 0.8 – 1.8% | 1.32 (0.81-2.14), p = 0.26 | |
| Hospital | 1 ^187^ | 1y | 31245 | 1% | 115696 | 1% | 1.03 (0.91-1.17), p = 0.64 | |
| **Heart failure** |  |  |  |  |  |  |  | |
| MI/PCI/CA | 4 ^164^ ^169^ ^173^ ^175^ | 1 – 4.4y | 1032 | 7.3 – 15.7% | 8680 | 4 – 9.9% | 1.91 (1.26-2.89), p < 0.01 | |
| Cardiac surgery | 3 ^156^ ^158^ ^188^ | 1 – 4.1y | 4519 | 9.2 – 10.2% | 25430 | 3 – 4.7% | 2.34 (1.87-2.93), p < 0.01 | |
| Major vascular surgery | - | - | - | - | - | - | - | |
| Other non-cardiac surgery | - | - | - | - | - | - | - | |
| ICU | 1 ^186^ | 3y | 4792 | 4.3% | 16764 | 1.9% | 2.24 (1.89-2.66), p < 0.01 | |
| Hospital | 3 ^187^ ^189^ ^190^ ^191^ ^192^ | 1 – 5y | 32447 | 4.6 – 20.8% | 116898 | 2.8 – 15.5% | 1.63 (1.54-1.73), p < 0.01 | |
| **Cardiovascular mortality** |  |  |  |  |  |  |  | |
| MI/PCI/CA | 4 ^152^ ^164^ ^193^ ^194^ | 1 – 5y | 4538 | 13.6 – 23.6% | 46127 | 1 – 8.2% | 4.26 (1.67-10.86), p < 0.01 | |
| Cardiac surgery | 3 ^154^ ^162^ ^174^ | 1 – 5.2y | 323 | 15.4 – 22.6% | 1542 | 6.4 – 10.4% | 1.9 (1.43-2.51), p < 0.01 | |
| Major vascular surgery | 1 ^195^ | 7y | 1801 | 29.5% | 1717 | 14.3% | 2.06 (1.8-2.36), p < 0.01 | |
| Other non-cardiac surgery | 2 ^196^ ^197^ | 5 – 10y | 27842 | 12.2 – 12.3% | 37854 | 6.2 – 8.1% | 1.73 (1.3-2.29), p < 0.01 | |
| ICU | 1 ^198^ | 5y | 838 | 12.5% | 1598 | 9.3% | 1.34 (1.06-1.7), p = 0.01 | |
| Hospital | - | - | - | - | - | - | - | |

Pooled estimates were obtained using a random-effects meta-analysis. AKI, acute kidney injury; CA, cardiac angiogram; FU, follow-up; HF, heart failure; ICU, intensive care unit; MI, myocardial infarction; PCI, percutaneous coronary intervention.

**Supplemental figures**

**Figure S1.** Sample size of included studies


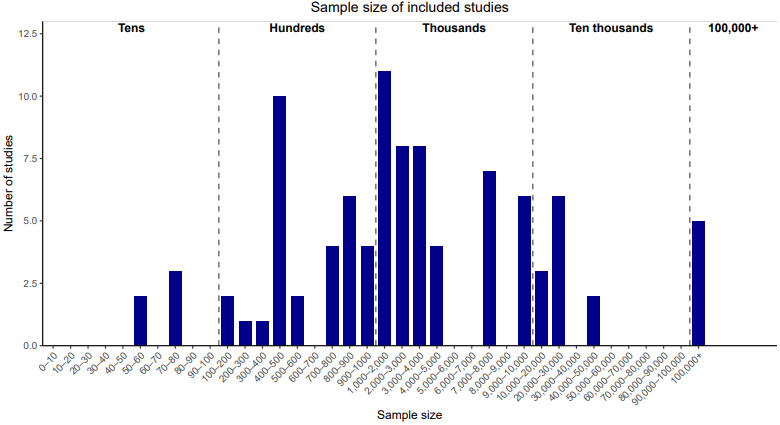


**Figures S2.** Countries of included studies

**
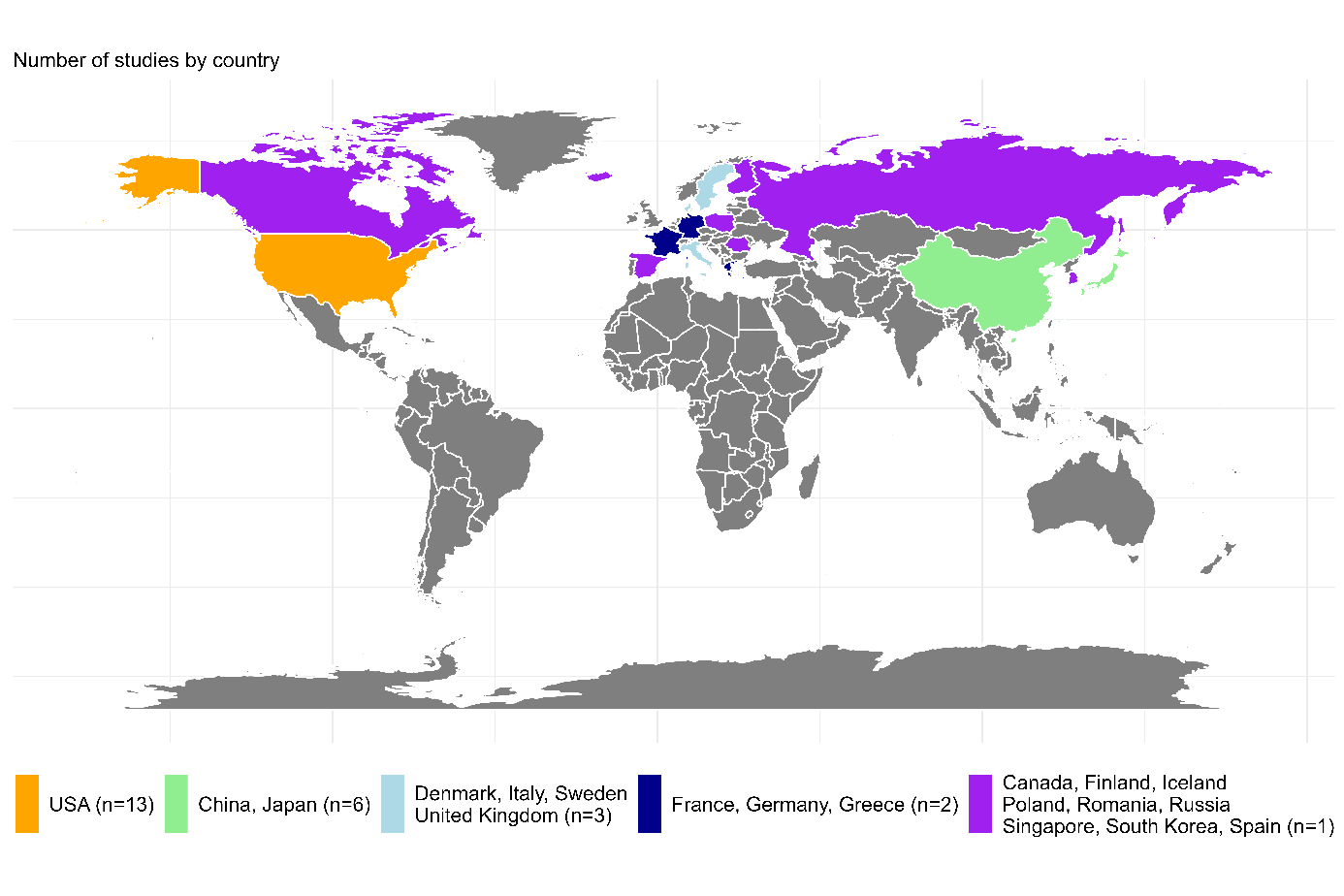
**

**Figure S3.** Funnel plots

**
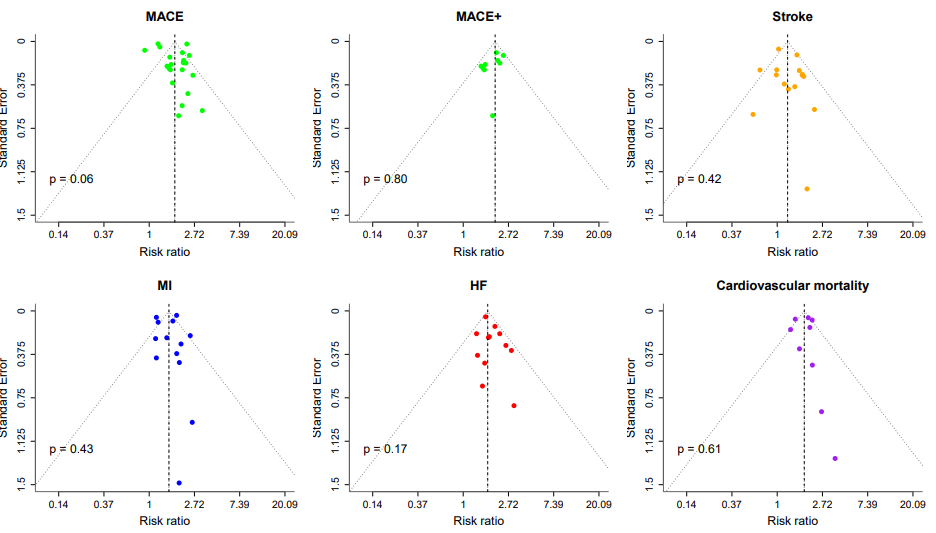
**

**Figure S4A-D.** Meta-regression plots

1. Heart failure


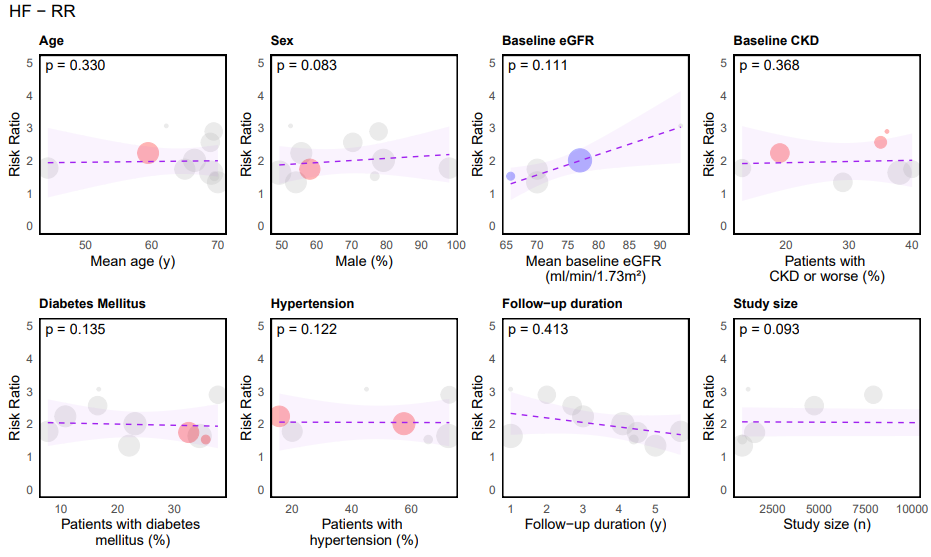


1. Myocardial infarction


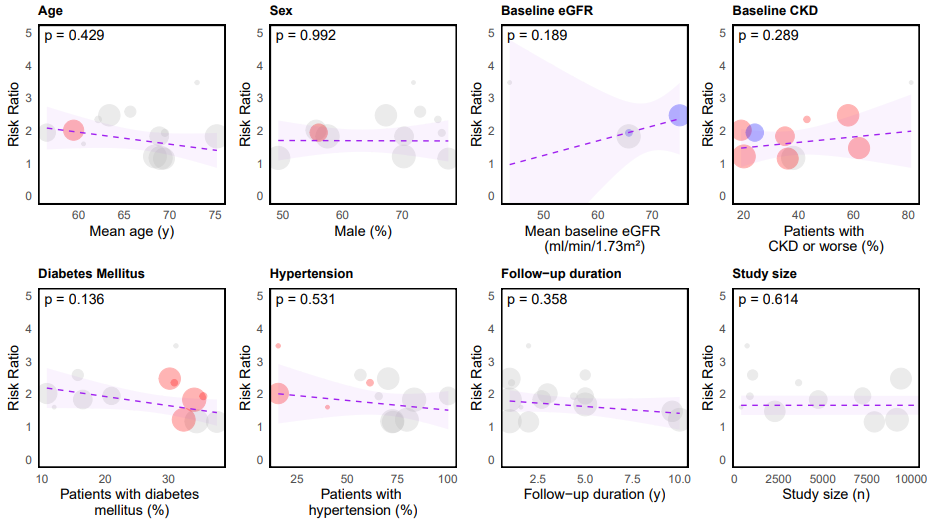


1. Cardiovascular mortality


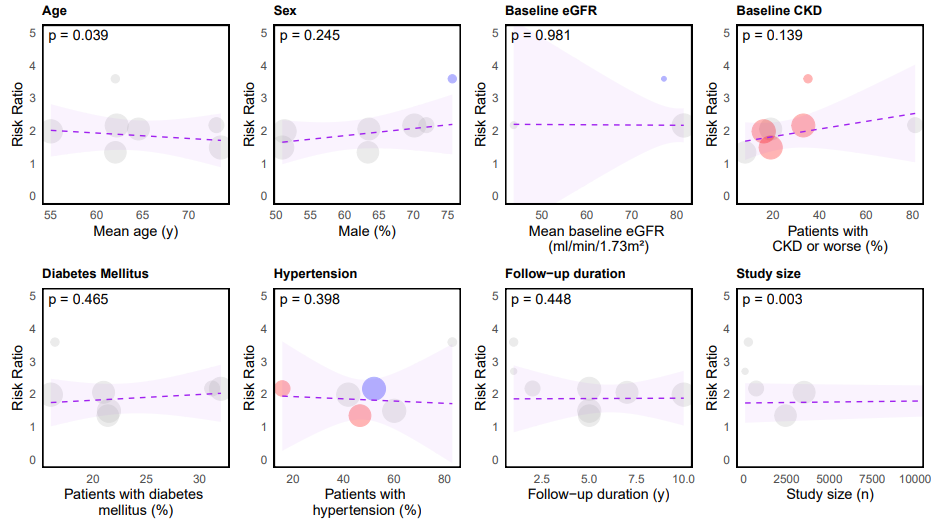


1. Stroke


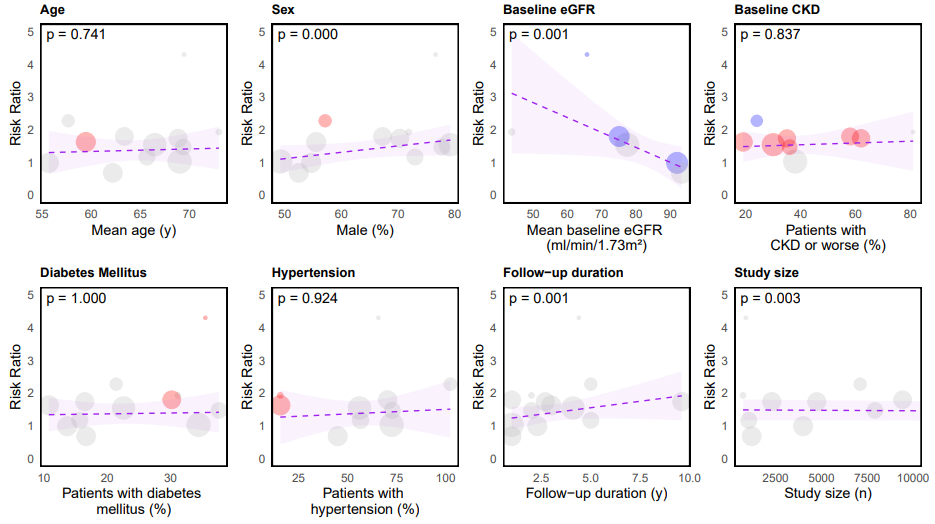


1. MACE+


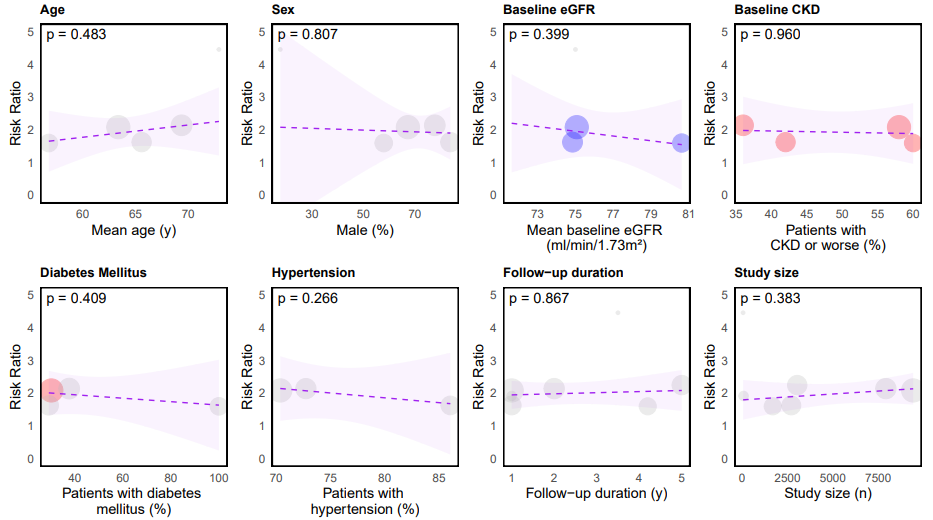


Every study is represented by a circle. Figure size indicates the study’s weight in the random effects model. The line indicates the regression line with 95% confidence intervals. Studies were included in the meta-regression analysis if they had a low or medium risk of bias. Blue and red indicate that the variable of interest was at least 10 percentage points lower or higher, respectively, in patients with AKI compared with patients without AKI (AKI as the reference group). eGFR, estimated glomerular filtration rate.

**
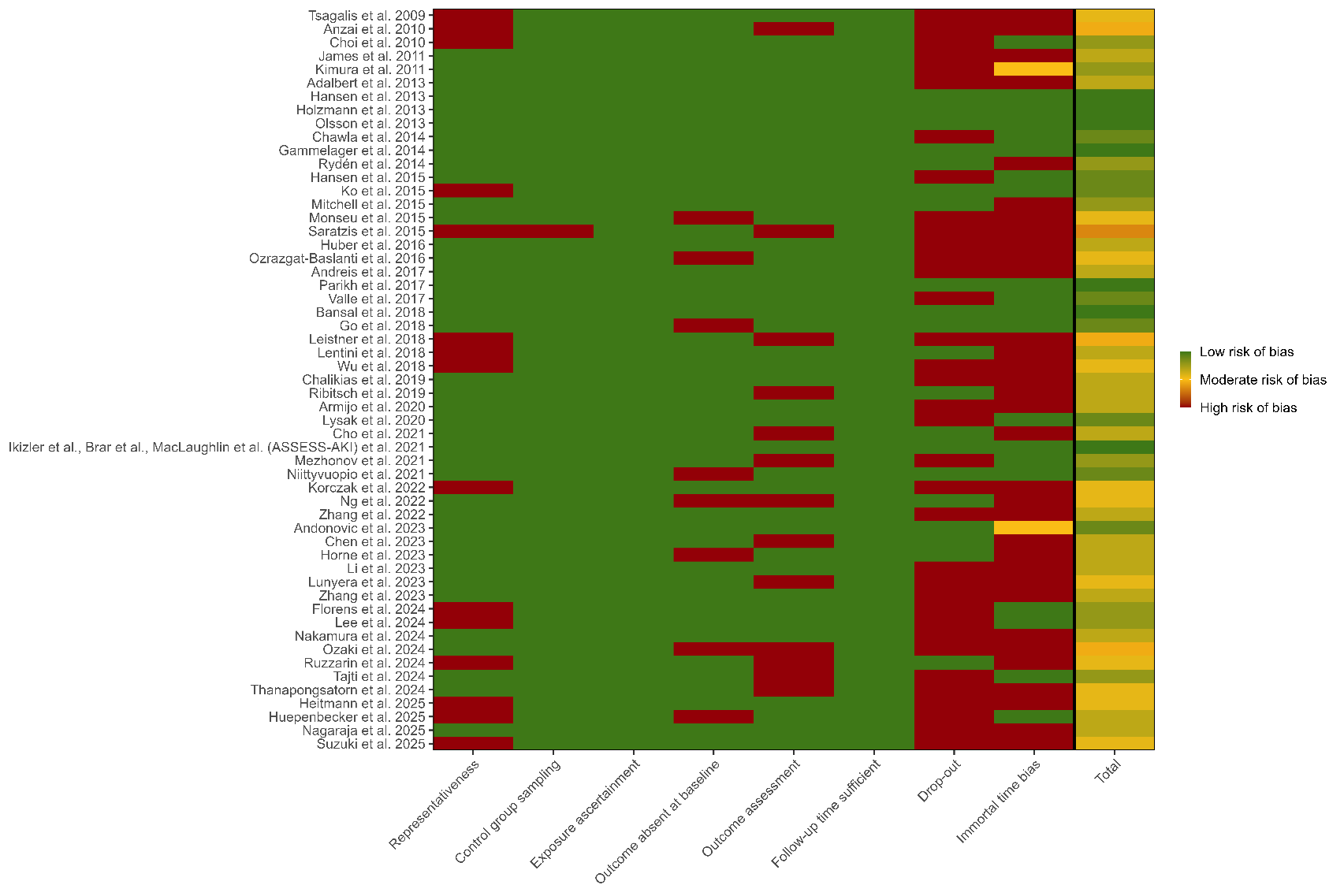
Figure S5.** Risk of bias assessment - Final rating per category

**Supplemental references**

1. Veltkamp DMJ, Porras CP, Gant CM, et al. Long-term risks of adverse kidney outcomes after acute kidney injury: a systematic review and meta-analysis. *Nephrol Dial Transplant*. Oct 30 2025;40(11):2143-2158. doi:10.1093/ndt/gfaf093

2. Omotoso BA, Abdel-Rahman EM, Xin W, et al. Acute kidney injury (AKI) outcome, a predictor of long-term major adverse cardiovascular events (MACE). *Clinical Nephrology*. 2016 2016;85(1):1-11. doi:doi:10.5414/CN108671

3. Mezhonov EM, Vyalkina YA, Vakulchik KA, Shalaev SV. Contrast-Induced Acute R enal Injury After Percutaneous Coronary InterventionI in Patients With ST-Segment Elevation Myocardial Infarction. *Kardiologiia*. 2018 2018;(8):5-11. doi:doi:doi:

4. Bouzas-Mosquera A, Vázquez-Rodríguez JM, Calviño-Santos R, et al. Contrast-induced nephropathy and acute renal failure following emergent cardiac catheterization: Incidence, risk factors and prognosis. *Revista Espanola de Cardiologia*. 2007 2007;60(10):1026-1034. doi:doi:10.1157/13111234

5. Marrani C, Zenjelaj T, Bartoli D, Corradi F, Innocenti R. Role of cystatin C as a biomarker of acute kidney injury and as an independent long-term predictor of cardiovascular events, mortality and functional outcome. *Italian Journal of Medicine*. 2012 2012;6(3):195-201. doi:doi:10.1016/j.itjm.2012.02.007

6. Kobalava ZD, Villeval'de SV, Gaskina AA, Maĭskov VV, Moiseev VS. Contrast-induced acute kidney injury after primary percutaneous coronary interventions: Prevalence, predictive factors, and outcomes. *Terapevticheskiĭ arkhiv*. 2015 2015;87(6):50-55. doi:doi:

7. Li XH, Xiao F, Zhang SY. [Investigation of risk factors of acute kidney injury after off-pump coronary artery bypass grafting and 3 years' follow-up]. *Beijing Da Xue Xue Bao Yi Xue Ban*. 2017-2-18 2017;49(1):131-6. doi:doi:

8. Kremneva LV, Suplotov SN. Risk factors and in-hospital outcomes of acute kidney injury that developed after coronary artery bypass grafting in patients with stable angina. *Ter Arkh*. 2018-9-20 2018;90(9):48-52. doi:doi:doi:10.26442/terarkh201890948-52

9. Kremneva LV, Gapon LI, Shalaev SV, Krasheninin DV. Acute Kidney Injury after Transcatheter Aortic Valve Implantation. *Rational Pharmacotherapy in Cardiology*. 2022 2022;18(3):261-267. doi:doi:doi:10.20996/1819-6446-2022-06-10

10. Khruleva YV, Efremovtseva MA, Timofeeva YA, Vatsik-Gorodetskaya MV, Kobalava ZD. Short- and long-term outcomes of patients with COVID-19 and acute kidney injury. *Cardiovascular Therapy and Prevention (Russian Federation)*. 2023 2023;22(6):25-34. doi:doi:doi:10.15829/1728-8800-2023-3587

11. Kremneva LV, Arutyunyan LA, Gapon LI, Suplotov SN, Shalaev SV. Acute kidney injury as a risk factor for atrial fibrillation after coronary artery bypass grafting — effects of sodium-glucose cotransporter-2 inhibitors. *Rational Pharmacotherapy in Cardiology*. 2023 2023;19(6):549-556. doi:doi:doi:10.20996/1819-6446-2023-2985

12. Welten GMJM, Chonchol M, Hoeks SE, et al. Statin therapy is associated with improved outcomes in vascular surgery patients with renal impairment. *American Heart Journal*. 2007 2007;154(5):954-961. doi:doi:10.1016/j.ahj.2007.06.040

13. Goldberg A, Kogan E, Hammerman H, Markiewicz W, Aronson D. The impact of transient and persistent acute kidney injury on long-term outcomes after acute myocardial infarction. *Kidney Int*. 2009-10 2009;76(8):900-6. doi:doi:10.1038/ki.2009.295

14. Hedley AJ, Roberts MA, Hayward PA, et al. Impact of Chronic Kidney Disease on Patient Outcome following Cardiac Surgery. *Heart Lung and Circulation*. 2010 2010;19(8):453-459. doi:doi:10.1016/j.hlc.2010.03.005

15. Orii K, Hioki M, Iedokoro Y, Shimizu K. Prognostic factors affecting clinical outcomes after coronary artery bypass surgery: Analysis of patients with chronic kidney disease after 5.9 years of follow-up. *Journal of Nippon Medical School*. 2011 2011;78(3):156-165. doi:doi:10.1272/jnms.78.156

16. Maioli M, Toso A, Leoncini M, Gallopin M, Musilli N, Bellandi F. Persistent renal damage after contrast-induced acute kidney injury: Incidence, evolution, risk factors, and prognosis. *Circulation*. 2012 2012;125(25):3099-3107. doi:doi:10.1161/CIRCULATIONAHA.111.085290

17. Ogita M, Sakakura K, Nakamura T, et al. Association between deteriorated renal function and long-term clinical outcomes after percutaneous coronary intervention. *Heart and Vessels*. 2012 2012;27(5):460-467. doi:doi:10.1007/s00380-011-0177-6

18. Ding FH, Lu L, Zhang RY, et al. Impact of elevated serum glycated albumin levels on contrast-induced acute kidney injury in diabetic patients with moderate to severe renal insufficiency undergoing coronary angiography. *International Journal of Cardiology*. 2013 2013;167(2):369-373. doi:doi:10.1016/j.ijcard.2011.12.101

19. Holzmann MJ, Gardell C, Jeppsson A, Sartipy U. Renal dysfunction and long-term risk of heart failure after coronary artery bypass grafting. *American Heart Journal*. 2013 2013;166(1):142-149.e1. doi:doi:10.1016/j.ahj.2013.03.005

20. James MT, Tonelli M, Ghali WA, et al. Renal outcomes associated with invasive versus conservative management of acute coronary syndrome: Propensity matched cohort study. *BMJ (Online)*. 2013 2013;347(7918)doi:doi:10.1136/bmj.f4151

21. Kume K, Yasuoka Y, Adachi H, et al. Impact of contrast-induced acute kidney injury on outcomes in patients with ST-segment elevation myocardial infarction undergoing primary percutaneous coronary intervention. *Cardiovasc Revasc Med*. 2013-9 2013;14(5):253-7. doi:doi:10.1016/j.carrev.2013.07.009

22. Currie A, Malietzis G, Askari A, et al. Impact of chronic kidney disease on postoperative outcome following colorectal cancer surgery. *Colorectal disease : the official journal of the Association of Coloproctology of Great Britain and Ireland*. 2014 2014;16(11):879-885. doi:doi:10.1111/codi.12665

23. Holzmann M, Jernberg T, Szummer K, Sartipy U. Long-term cardiovascular outcomes in patients with chronic kidney disease undergoing coronary artery bypass graft surgery for acute coronary syndromes. *Journal of the American Heart Association*. 2014 2014;3(2)doi:doi:10.1161/JAHA.113.000707

24. Watabe H, Sato A, Hoshi T, et al. Association of contrast-induced acute kidney injury with long-term cardiovascular events in acute coronary syndrome patients with chronic kidney disease undergoing emergent percutaneous coronary intervention. *International Journal of Cardiology*. 2014 2014;174(1):57-63. doi:doi:10.1016/j.ijcard.2014.03.146

25. Wu VC, Wu CH, Huang TM, et al. Long-term risk of coronary events after AKI. *J Am Soc Nephrol*. 2014-3 2014;25(3):595-605. doi:doi:10.1681/asn.2013060610

26. Wu VC, Wu PC, Wu CH, et al. The impact of acute kidney injury on the long-term risk of stroke. *Journal of the American Heart Association*. 2014 2014;3(4)doi:doi:10.1161/JAHA.114.000933

27. Arslan S, Abaci, O., Koças, C., Koçaş, B.B., Çetinkal, G., Dalgiç, Y., Karaca, O.S., Yildiz, A., Doǧan, S.M. Long-term follow- up of patients who developed contrast-induced acute kidney injury in patients with acute coronary syndrome. 2015;

28. Chan W, Ivanov J, Ko D, et al. Clinical outcomes of treatment by percutaneous coronary intervention versus coronary artery bypass graft surgery in patients with chronic kidney disease undergoing index revascularization in Ontario. *Circ Cardiovasc Interv*. 2015-1 2015;8(1)doi:doi:10.1161/circinterventions.114.001973

29. Sato A, Hoshi T, Kakefuda Y, et al. Effect of the Mehran risk score for the prediction of clinical outcomes after percutaneous coronary intervention. *J Cardiol*. 2015-11 2015;66(5):417-22. doi:doi:10.1016/j.jjcc.2014.12.016

30. Attizzani GF, Ohno Y, Latib A, et al. Age-Related Differences in 1- and 12-Month Outcomes in Patients Undergoing Transcatheter Aortic Valve Implantation (from a Large Multicenter Data Repository). *Am J Cardiol*. 2016-10-1 2016;118(7):1024-30. doi:doi:10.1016/j.amjcard.2016.07.018

31. Brown JR, Solomon RJ, Robey RB, et al. Chronic Kidney Disease Progression and Cardiovascular Outcomes Following Cardiac Catheterization-A Population-Controlled Study. *J Am Heart Assoc*. 2016-10-14 2016;5(10)doi:doi:10.1161/jaha.116.003812

32. Park SD, Moon J, Kwon SW, et al. Prognostic impact of combined contrast-induced acute kidney injury and hypoxic liver injury in patients with ST elevation myocardial infarction undergoing primary percutaneous coronary intervention: Results from INTERSTELLAR registry. *Plos One*. 2016 2016;11(7)doi:doi:10.1371/journal.pone.0159416

33. Tonkonogi A, Carlsson AC, Helmersson-Karlqvist J, Larsson A, Ärnlöv J. Associations between urinary kidney injury biomarkers and cardiovascular mortality risk in elderly men with diabetes. *Ups J Med Sci*. 2016-8 2016;121(3):174-8. doi:doi:10.1080/03009734.2016.1192704

34. Fortrie G, Manintveld OC, Constantinescu AA, van de Woestijne PC, Betjes MGH. Renal function at 1 year after cardiac transplantation rather than acute kidney injury is highly associated with long-term patient survival and loss of renal function – a retrospective cohort study. *Transplant International*. 2017 2017;30(8):788-798. doi:doi:10.1111/tri.12940

35. Nakada Y, Kawakami R, Matsui M, et al. Prognostic value of urinary neutrophil gelatinase-associated lipocalin on the first day of admission for adverse events in patients with acute decompensated heart failure. *Journal of the American Heart Association*. 2017 2017;6(5)doi:doi:10.1161/JAHA.116.004582

36. Nakahashi H, Kosuge M, Sakamaki K, et al. Combined impact of chronic kidney disease and contrast-induced nephropathy on long-term outcomes in patients with ST-segment elevation acute myocardial infarction who undergo primary percutaneous coronary intervention. *Heart Vessels*. 2017-1 2017;32(1):22-29. doi:doi:10.1007/s00380-016-0836-8

37. Sato A, Aonuma K, Watanabe M, et al. Association of contrast-induced nephropathy with risk of adverse clinical outcomes in patients with cardiac catheterization: From the CINC-J study. *Int J Cardiol*. 2017-1-15 2017;227:424-429. doi:doi:10.1016/j.ijcard.2016.11.019

38. Shih CJ, Chao PW, Ou SM, Chen YT. Long-Term Risk of Cardiovascular Events in Patients With Chronic Kidney Disease Who Have Survived Sepsis: A Nationwide Cohort Study. *J Am Heart Assoc*. 2017-2-10 2017;6(2)doi:doi:10.1161/jaha.116.004613

39. Uzunhasan I, Yildiz A, Arslan S, et al. Contrast-Induced Acute Kidney Injury Is Associated with Long-Term Adverse Events in Patients with Acute Coronary syndrome. *Angiology*. 2017 2017;68(7):621-626. doi:doi:10.1177/0003319716676173

40. Ribitsch W, Horina JH, Quehenberger F, Rosenkranz AR, Schilcher G. Contrast Induced Acute Kidney Injury and its Impact on Mid-Term Kidney Function, Cardiovascular Events and Mortality. *Sci Rep-Uk*. Nov 15 2019;9doi:ARTN 16896

10.1038/s41598-019-53040-5

41. Pinier C, Gatault P, Fauchier L, et al. Specific impact of past and new major cardiovascular events on acute kidney injury and end-stage renal disease risks in diabetes: a dynamic view. *Clinical Kidney Journal*. Feb 2020;13(1):17-23. doi:10.1093/ckj/sfz028

42. Adachi Y, Yamamoto M, Shimura T, et al. Late Adverse Cardiorenal Events of Catheter Procedure-Related Acute Kidney Injury After Transcatheter Aortic Valve Implantation. *Am J Cardiol*. Oct 15 2020;133:89-97. doi:10.1016/j.amjcard.2020.07.041

43. Peillex M, Marchandot B, Matsushita K, et al. Acute kidney injury and acute kidney recovery following Transcatheter Aortic Valve Replacement. *Plos One*. 2021;16(8)doi:ARTN e0255806

10.1371/journal.pone.0255806

44. Skalsky K, Shiyovich A, Bental T, et al. Temporal trends of acute kidney injury in patients undergoing percutaneous coronary intervention over a span of 12 years. *International Journal of Cardiology*. Mar 1 2021;326:44-48. doi:10.1016/j.ijcard.2020.10.039

45. Zhu J, Liu W. Acute kidney injury predicts poor left ventricular function for patients with peripartum cardiomyopathy. *BMC Cardiovascular Disorders*. 2021 2021;21(1)doi:doi:doi:10.1186/s12872-021-02021-6

46. Mohebi R, Karimi Galougahi K, Garcia JJ, et al. Long-Term Clinical Impact of Contrast-Associated Acute Kidney Injury Following PCI: An ADAPT-DES Substudy. *JACC Cardiovasc Interv*. Apr 11 2022;15(7):753-766. doi:10.1016/j.jcin.2021.11.026

47. Lu JY, Boparai MS, Shi C, et al. Long-term outcomes of COVID-19 survivors with hospital AKI: association with time to recovery from AKI. *Nephrology, dialysis, transplantation : official publication of the European Dialysis and Transplant Association - European Renal Association*. 2023 2023;doi:doi:doi:10.1093/ndt/gfad020

48. Zhang XY, Fan ZG, Xu HM, Xu K, Tian NL. Clinical Outcomes for Acute Kidney Injury in Acute Myocardial Infarction Patients after Intra-Aortic Balloon Pump Implantation: A Single-Center Observational Study. *Rev Cardiovasc Med*. Jun 2023;24(6)doi:ARTN 172

10.31083/j.rcm2406172

49. Presume J, Cunha GJL, Rocha BML, et al. Acute kidney injury patterns in acute heart failure: The prognostic value of worsening renal function and its timing. *Rev Port Cardiol*. May 2023;42(5):423-430. doi:10.1016/j.repc.2022.06.015

50. Marques da Silva B, Gameiro J, Lei Teixeira J, et al. Long-term outcomes after AKI in hospitalized patients with COVID-19. *Nefrologia*. 2024 2024;doi:doi:doi:10.1016/j.nefro.2024.08.003

51. Thanapongsatorn P, Tanomchartchai A, Assavahanrit J. Long-term outcomes of acute kidney injury in acute decompensated heart failure: identifying true cardiorenal syndrome and unveiling prognostic significance. *Kidney Res Clin Pract*. Jul 2024;43(4):480-491. doi:10.23876/j.krcp.23.323

52. Tyl B, Costa A, Vora P, et al. Association of a history of acute kidney injury with major adverse cardiovascular events in chronic kidney disease patients. *European Heart Journal*. 2024;45(Supplement_1)doi:10.1093/eurheartj/ehae666.3259

53. Khan AA, Hasan, H., Khan, A., Lopez, D.M., Arany, Z.P., Sabe, M.A., Wu, W.-C.H., Choudhary, G., Imran, T.F. ACUTE KIDNEY INJURY AT DIAGNOSIS AND LONG-TERM ADVERSE CARDIOVASCULAR OUTCOMES IN PERIPARTUM CARDIOMYOPATHY. 2025;

54. Yang TY, Chuang MH, Lin HM, et al. Clinical outcomes after immune checkpoint inhibitor-associated acute kidney injury: a cohort study. *Bmj Open*. Feb 7 2025;15(2)doi:ARTN e092752

10.1136/bmjopen-2024-092752

55. Go AS, Parikh CR, Ikizler TA, et al. The assessment, serial evaluation, and subsequent sequelae of acute kidney injury (ASSESS-AKI) study: design and methods. *BMC Nephrol*. 2010-8-27 2010;11:22. doi:doi:10.1186/1471-2369-11-22

56. Narula A, Mehran R, Weisz G, et al. Contrast-induced acute kidney injury after primary percutaneous coronary intervention: Results from the HORIZONS-AMI substudy. *European Heart Journal*. 2014 2014;35(23):1533-1540. doi:doi:10.1093/eurheartj/ehu063

57. Tsai HS, Chen YC, Chu PH. The Influence of Acute Kidney Injury on Acute Cardiovascular Disease. *Acta Cardiol Sin*. 2014-3 2014;30(2):93-7. doi:doi:

58. Crimi G, Leonardi S, Costa F, et al. Incidence, prognostic impact, and optimal definition of contrast-induced acute kidney injury in consecutive patients with stable or unstable coronary artery disease undergoing percutaneous coronary intervention. insights from the all-comer PRODIGY trial. *Catheterization and Cardiovascular Interventions*. 2015 2015;86(1):E19-E27. doi:doi:10.1002/ccd.25822

59. Giacoppo D, Madhavan MV, Baber U, et al. Impact of Contrast-Induced Acute Kidney Injury After Percutaneous Coronary Intervention on Short- and Long-Term Outcomes: Pooled Analysis From the HORIZONS-AMI and ACUITY Trials. *Circ Cardiovasc Interv*. 2015-8 2015;8(8):e002475. doi:doi:10.1161/circinterventions.114.002475

60. Usmiani T, Andreis A, Budano C, et al. AKIGUARD (Acute Kidney Injury GUARding Device) trial: in-hospital and one-year outcomes. *J Cardiovasc Med (Hagerstown)*. 2016-7 2016;17(7):530-7. doi:doi:10.2459/jcm.0000000000000348

61. Warren J, Mehran R, Baber U, et al. Incidence and impact of acute kidney injury in patients with acute coronary syndromes treated with coronary artery bypass grafting: Insights from the Harmonizing Outcomes With Revascularization and Stents in Acute Myocardial Infarction (HORIZONS-AMI) and Acute Catheterization and Urgent Intervent. *American Heart Journal*. 2016 2016;171(1):40-47. doi:doi:10.1016/j.ahj.2015.07.001

62. Geri G, Stengel B, Jacquelinet C, et al. Prediction of chronic kidney disease after acute kidney injury in ICU patients: study protocol for the PREDICT multicenter prospective observational study. *Annals of Intensive Care*. 2018 2018;8(1)doi:doi:doi:10.1186/s13613-018-0421-7

63. Arbel Y, Fuster V, Baber U, Hamza TH, Siami FS, Farkouh ME. Incidence, determinants and impact of acute kidney injury in patients with diabetes mellitus and multivessel disease undergoing coronary revascularization: Results from the FREEDOM trial. *Int J Cardiol*. Oct 15 2019;293:197-202. doi:10.1016/j.ijcard.2019.05.064

64. Dieter BP, Daratha KB, McPherson SM, Short R, Alicic RZ, Tuttle KR. Association of Acute Kidney Injury with Cardiovascular Events and Death in Systolic Blood Pressure Intervention Trial. *American Journal of Nephrology*. 2019;49(5):359-367. doi:10.1159/000499574

65. De Rosa R, Morici N, De Servi S, et al. Impact of renal dysfunction and acute kidney injury on outcome in elderly patients with acute coronary syndrome undergoing percutaneous coronary intervention. *Eur Heart J Acute Cardiovasc Care*. Dec 18 2021;10(10):1160-1169. doi:10.1177/2048872620920475

66. Schytz PA, Blanche P, Nissen AB, et al. Acute kidney injury and risk of cardiovascular outcomes: A nationwide cohort study. *Nefrologia*. May-Jun 2022;42(3):338-346. doi:10.1016/j.nefro.2021.06.007

67. Kaneda K, Shiomi H, Abe M, et al. Post-contrast Acute Kidney Injury After Emergent and Elective Percutaneous Coronary Intervention (from the CREDO-Kyoto PCI/CABG Registry Cohort 3). *American Journal of Cardiology*. Sep 1 2023;202:58-66. doi:10.1016/j.amjcard.2023.06.031

68. Landi A, Branca M, Leonardi S, et al. Transient vs In-Hospital Persistent Acute Kidney Injury in Patients With Acute Coronary Syndrome. *Jacc-Cardiovasc Inte*. Jan 23 2023;16(2):193-205. doi:10.1016/j.jcin.2022.10.009

69. Sesso R, Roque A, Vicioso B, Stella S. Prognosis of ARF in hospitalized elderly patients. *American Journal of Kidney Diseases*. 2004 2004;44(3):410-419. doi:doi:10.1053/j.ajkd.2004.05.022

70. Parikh CR, Coca SG, Wang Y, Masoudi FA, Krumholz HM. Long-term prognosis of acute kidney injury after acute myocardial infarction. *Archives of Internal Medicine*. 2008 2008;168(9):987-995. doi:doi:10.1001/archinte.168.9.987

71. Hobson CE, Yavas S, Segal MS, et al. Acute kidney injury is associated with increased long-term mortality after cardiothoracic surgery. *Circulation*. 2009 2009;119(18):2444-2453. doi:doi:10.1161/CIRCULATIONAHA.108.800011

72. Brown JR, Kramer RS, Coca SG, Parikh CR. Duration of acute kidney injury impacts long-term survival after cardiac surgery. *Ann Thorac Surg*. 2010-10 2010;90(4):1142-8. doi:doi:10.1016/j.athoracsur.2010.04.039

73. Ishibashi Y, Yamauchi M, Musha H, Mikami T, Kawasaki K, Miyake F. Impact of contrast-induced nephropathy and cardiovascular events by serum cystatin C in renal insufficiency patients undergoing cardiac catheterization. *Angiology*. 2010 2010;61(8):724-730. doi:doi:

74. Kim MJ, Choi HS, Oh SH, et al. Impact of acute kidney injury on clinical outcomes after ST elevation acute myocardial infarction. *Yonsei Medical Journal*. 2011 2011;52(4):603-609. doi:doi:10.3349/ymj.2011.52.4.603

75. Wi J, Ko YG, Kim JS, et al. Impact of contrast-induced acute kidney injury with transient or persistent renal dysfunction on long-term outcomes of patients with acute myocardial infarction undergoing percutaneous coronary intervention. *Heart*. 2011 2011;97(21):1753-1757. doi:doi:10.1136/hrt.2010.218677

76. Brito GA, Balbi AL, Abrão JM, Ponce D. Long-term outcome of patients followed by nephrologists after an acute tubular necrosis episode. *Int J Nephrol*. 2012 2012;2012:361528. doi:doi:10.1155/2012/361528

77. Bruetto RG, Rodrigues FB, Torres US, Otaviano AP, Zanetta DMT, Burdmann EA. Renal function at hospital admission and mortality due to acute kidney injury after myocardial infarction. *Plos One*. 2012 2012;7(4)doi:doi:10.1371/journal.pone.0035496

78. Zhou Q, Zhao C, Xie D, et al. Acute and acute-on-chronic kidney injury of patients with decompensated heart failure: impact on outcomes. *BMC Nephrol*. 2012-7-2 2012;13:51. doi:doi:10.1186/1471-2369-13-51

79. Choi JS, Kim YA, Kim MJ, et al. Relation between transient or persistent acute kidney injury and long-term mortality in patients with myocardial infarction. *Am J Cardiol*. 2013-7-1 2013;112(1):41-5. doi:doi:10.1016/j.amjcard.2013.02.051

80. Hsieh MJ, Chen YC, Chen CC, Wang CL, Wu LS, Wang CC. Renal dysfunction on admission, worsening renal function, and severity of acute kidney injury predict 2-year mortality in patients with acute myocardial infarction. *Circulation Journal*. 2013 2013;77(1):217-223. doi:doi:10.1253/circj.CJ-12-0539

81. Lopez-Delgado JC, Esteve F, Torrado H, et al. Influence of acute kidney injury on short- and long-term outcomes in patients undergoing cardiac surgery: Risk factors and prognostic value of a modified RIFLE classification. *Critical Care*. 2013 2013;17(6)doi:doi:10.1186/cc13159

82. Neyra JA, Shah S, Mooney R, Jacobsen G, Yee J, Novak JE. Contrast-induced acute kidney injury following coronary angiography: a cohort study of hospitalized patients with or without chronic kidney disease. *Nephrol Dial Transplant*. 2013-6 2013;28(6):1463-71. doi:doi:10.1093/ndt/gft082

83. Pannu N, James M, Hemmelgarn B, Klarenbach S. Association between AKI, recovery of renal function, and long-term outcomes after hospital discharge. *Clinical Journal of the American Society of Nephrology*. 2013 2013;8(2):194-202. doi:doi:10.2215/CJN.06480612

84. Ivert T, Holzmann MJ, Sartipy U. Survival in patients with acute kidney injury requiring dialysis after coronary artery bypass grafting. *European Journal of Cardio-thoracic Surgery*. 2014 2014;45(2):312-317. doi:doi:10.1093/ejcts/ezt247

85. Wonnacott A, Meran S, Amphlett B, Talabani B, Phillips A. Epidemiology and outcomes in community-acquired versus hospital-acquired aki. *Clinical Journal of the American Society of Nephrology*. 2014 2014;9(6):1007-1014. doi:doi:10.2215/CJN.07920713

86. Abaci O, Arat Ozkan A, Kocas C, et al. Impact of Rosuvastatin on Contrast-Induced Acute Kidney Injury in Patients at High Risk for Nephropathy Undergoing Elective Angiography. *American Journal of Cardiology*. 2015 2015;115(7):867-871. doi:doi:10.1016/j.amjcard.2015.01.007

87. Geri G, Guillemet L, Dumas F, et al. Acute kidney injury after out-of-hospital cardiac arrest: risk factors and prognosis in a large cohort. *Intensive Care Medicine*. 2015 2015;41(7):1273-1280. doi:doi:10.1007/s00134-015-3848-4

88. Saratzis A, Melas N, Mahmood A, Sarafidis P. Incidence of Acute Kidney Injury (AKI) after Endovascular Abdominal Aortic Aneurysm Repair (EVAR) and impact on outcome. *European Journal of Vascular and Endovascular Surgery*. 2015 2015;49(5):534-540. doi:doi:10.1016/j.ejvs.2015.01.002

89. Xu JR, Zhu JM, Jiang J, et al. Risk Factors for Long-Term Mortality and Progressive Chronic Kidney Disease Associated with Acute Kidney Injury after Cardiac Surgery. *Medicine (United States)*. 2015 2015;94(45):e2025. doi:doi:10.1097/MD.0000000000002025

90. Zhang W, Wen D, Zou YF, et al. One-year survival and renal function recovery of acute kidney injury patients with chronic heart failure. *CardioRenal Medicine*. 2015 2015;5(1):40-47. doi:doi:10.1159/000369834

91. Centola M, Lucreziotti S, Salerno-Uriarte D, et al. A COMPARISON BETWEEN TWO DIFFERENT DEFINITIONS OF CONTRAST-INDUCED ACUTE KIDNEY INJURY IN PATIENTS WITH ST-SEGMENT ELEVATION MYOCARDIAL INFARCTION UNDERGOING PRIMARY PERCUTANEUOS CORONARY INTERVENTION. *Journal of the American College of Cardiology*. 2016/04/05/ 2016;67(13, Supplement):86. doi:<https://doi.org/10.1016/S0735-1097(16)30087-0>

92. Santana-Santos E, Kamei FK, do Nascimento TK, et al. Long-Term Follow-Up Evaluation of Renal Function in Patients with Chronic Kidney Disease Undergoing Cardiac Surgery. *Int J Nephrol*. 2016 2016;2016:9680718. doi:doi:10.1155/2016/9680718

93. Wang X, Ren HM, Hu CY, et al. Predictors and in-hospital outcomes of preoperative acute kidney injury in patients with type A acute aortic dissection. *J Geriatr Cardiol*. 2016-8 2016;13(8):679-684. doi:doi:10.11909/j.issn.1671-5411.2016.08.006

94. Gupta T, Goel K, Kolte D, et al. Association of Chronic Kidney Disease With In-Hospital Outcomes of Transcatheter Aortic Valve Replacement. *JACC: Cardiovascular Interventions*. 2017 2017;10(20):2050-2060. doi:doi:10.1016/j.jcin.2017.07.044

95. Kuji S, Kosuge M, Kimura K, et al. Impact of acute kidney injury on in-hospital outcomes of patients with acute myocardial infarction: Results from the japanese registry of acute myocardial infarction diagnosed by Universal Definition (J-MINUET) substudy. *Circulation Journal*. 2017 2017;81(5):733-739. doi:doi:10.1253/circj.CJ-16-1094

96. Sawhney S, Marks A, Fluck N, Levin A, Prescott G, Black C. Intermediate and Long-term Outcomes of Survivors of Acute Kidney Injury Episodes: A Large Population-Based Cohort Study. *American Journal of Kidney Diseases*. 2017 2017;69(1):18-28. doi:doi:10.1053/j.ajkd.2016.05.018

97. Wu B, Ma L, Shao Y, et al. Effect of Cardiac Surgery-Associated Acute Kidney Injury on Long-Term Outcomes of Chinese Patients: A Historical Cohort Study. *Blood Purification*. 2017 2017;44(3):227-233. doi:doi:10.1159/000478967

98. Pourafkari L, Arora P, Porhomayon J, Dosluoglu HH, Arora P, Nader ND. Acute kidney injury after non-cardiovascular surgery: risk factors and impact on development of chronic kidney disease and long-term mortality. *Curr Med Res Opin*. 2018;34(10):1829-1837. doi:10.1080/03007995.2018.1459527

99. Khatua CR, Sahu SK, Barik RK, et al. Validation of International Club of Ascites subclassification of stage 1 acute kidney injury in chronic liver disease. *JGH Open*. 2019 2019;3(4):290-294. doi:doi:doi:10.1002/jgh3.12152

100. Kofman N, Margolis G, Gal-Oz A, et al. Long-term renal outcomes and mortality following renal injury among myocardial infarction patients treated by primary percutaneous intervention. *Coronary Artery Disease*. 2019 2019;30(2):87-92. doi:doi:doi:10.1097/mca.0000000000000678

101. Walther CP, Winkelmayer WC, Niu J, et al. Acute Kidney Injury With Ventricular Assist Device Placement: National Estimates of Trends and Outcomes. *Am J Kidney Dis*. 2019-11 2019;74(5):650-658. doi:doi:doi:10.1053/j.ajkd.2019.03.423

102. Hertzberg D, Holzmann MJ, Than M, Pickering JW. Acute kidney injury in patients presenting with chest pain to the emergency department, a descriptive study of the most common discharge diagnoses and mortality. *Eur J Emerg Med*. Aug 2019;26(4):242-248. doi:10.1097/Mej.0000000000000552

103. Holgado JL, Lopez C, Fern, et al. Acute kidney injury in heart failure: a population study. *ESC Heart Failure*. 2020 2020;7(2):415-422. doi:doi:doi:10.1002/ehf2.12595

104. Josa-Laorden C, Giménez-López I, Rubio-Gracia J, Garcés Horna V, Sánchez-Marteles M, Pérez-Calvo JI. Prognostic significance of acute kidney injury and small increases in creatinine concentration during acute decompensation of heart failure. *Revista Clinica Espanola*. 2020 2020;220(9):561-568. doi:doi:doi:10.1016/j.rce.2019.11.008

105. Khoury S, Margolis G, Ravid D, Rozenbaum Z, Keren G, Shacham Y. Outcomes of early and reversible renal impairment in patients with ST segment elevation myocardial infarction undergoing percutaneous coronary intervention. *Eur Heart J-Acute Ca*. Oct 2020;9(7):684-689. doi:10.1177/2048872618808456

106. Lei L, Xue Y, Guo ZD, et al. A comparison between different definitions of contrast-induced acute kidney injury for long-term mortality in patients with acute myocardial infarction. *Ijc Heart Vasc*. Jun 2020;28doi:ARTN 100522

10.1016/j.ijcha.2020.100522

107. Merdler I, Loewenstein I, Zahler D, et al. Acute cardiorenal anemia syndrome among ST-elevation myocardial infarction patients treated by primary percutaneous intervention. *Coron Artery Dis*. 2021-6-1 2021;32(4):275-280. doi:doi:doi:10.1097/mca.0000000000000973

108. Crimi G, De Marzo V, De Marco F, et al. Acute Kidney Injury After Transcatheter Aortic Valve Replacement Mediates the Effect of Chronic Kidney Disease. *Journal of the American Heart Association*. Oct 4 2022;11(19)doi:ARTN e024589

10.1161/JAHA.121.024589

109. Fu AR, Liao J, Wang W. The Relationship between the Risk Factors of Ci-Aki and Mace and the Cys C Level in Elderly Patients after Pci. *Acta Medica Mediterr*. 2022;38(1):529-533. doi:10.19193/0393-6384_2022_1_84

110. Huckaby LV, Seese LM, Hess N, et al. Fate of the Kidneys in Patients with Post-Operative Renal Failure After Cardiac Surgery. *J Surg Res*. Apr 2022;272:166-174. doi:10.1016/j.jss.2021.08.025

111. Luo X, Yan P, Zhang N, et al. Early recovery status and outcomes after sepsis-associated acute kidney injury in critically ill patients. *Zhong Nan Da Xue Xue Bao Yi Xue Ban*. 2022-5-28 2022;47(5):535-545. doi:doi:doi:10.11817/j.issn.1672-7347.2022.210368 %(危重症患者脓毒症急性肾损伤后的早期恢复模式与预后.

112. Shemiesa RS, Abdelsalama M, Elnagara SS, Mohameda AH, Sayed-Ahmeda N, Tawfika M. Characteristics, risk factors and outcomes of community-acquired acute kidney injury in the elderly: a prospective tertiary hospital study, Egypt. *African Health Sciences*. 2022 2022;22(2):350-361. doi:doi:doi:10.4314/ahs.v22i2.40

113. Tanaka T, Kavsur R, Sugiura A, et al. Acute Kidney Injury Following Tricuspid Transcatheter Edge-to-Edge Repair. *Jacc-Cardiovasc Inte*. Oct 10 2022;15(19):1936-1945. doi:10.1016/j.jcin.2022.07.018

114. Mutyala SCVS, Vaggar JN, Borker S, et al. Acute Kidney Injury among Post Cardiac Surgery Patients: A Retrospective Study. *Journal of the Indian Medical Association*. 2023 2023;121(3):15-20. doi:doi:doi:

115. Tarar ZI, Zafar MU, Farooq U, et al. Effect of acute kidney injury on hospital-based outcomes in patients admitted for variceal hemorrhage. Analysis of national inpatient sample database. *Przeglad Gastroenterologiczny*. 2023 2023;18(1):85-92. doi:doi:doi:10.5114/pg.2022.115135

116. Chen ZY, Zhou DL, Jiang YB, et al. Contrast-associated acute kidney injury in myocardial infarction patients undergoing elective percutaneous coronary intervention: insight from the Iodixanol-AKI Registry. *Intern Emerg Med*. Oct 2024;19(7):1859-1866. doi:10.1007/s11739-024-03673-w

117. Choi UE, Hepner DL, Connors JM. Acute Kidney Injury after Cardiac Surgery Is Associated with Increased Iron Deficiency Anemia Risk. *Blood*. 2024;144(Supplement 1):7699-7699. doi:10.1182/blood-2024-208183

118. Kobalava ZD, Ilyinichna KN, Vladimirovna TV, Vazgenovna KL. Prognostic Value of Acute Kidney Injury in Patients Hospitalized with Acute Decompensation of Chronic Heart Failure. *Annals of Clinical Cardiology*. 2024;6(2):77-81. doi:10.4103/accj.Accj_14_24

119. Cioffi G, Tarantini L, Pulignano G, et al. Prevalence, predictors and prognostic value of acute impairment in renal function during intensive unloading therapy in a community population hospitalized for decompensated heart failure. *Journal of Cardiovascular Medicine*. 2007 2007;8(6):419-427. doi:doi:10.2459/01.JCM.0000269715.95317.33

120. Szczech LA, Granger CB, Dasta JF, et al. Acute kidney injury and cardiovascular outcomes in acute severe hypertension. *Circulation*. 2010-5-25 2010;121(20):2183-91. doi:doi:10.1161/circulationaha.109.896597

121. Schnabel RB, Seiffert M, Wilde S, et al. Kidney injury and mortality after transcatheter aortic valve implantation in a routine clinical cohort. *Catheter Cardiovasc Interv*. 2015-2-15 2015;85(3):440-7. doi:doi:10.1002/ccd.25588

122. Zhao XJ, Zhu FX, Li S, Zhang HB, An YZ. Acute kidney injury is an independent risk factor for myocardial injury after noncardiac surgery in critical patients. *Journal of Critical Care*. 2017 2017;39:225-231. doi:doi:10.1016/j.jcrc.2017.01.011

123. Shiyovich A, Skalsky K, Steinmetz T, et al. Acute Kidney Injury Following Admission with Acute Coronary Syndrome: The Role of Diabetes Mellitus. *J Clin Med*. Nov 2021;10(21)doi:ARTN 4931

10.3390/jcm10214931

124. Wang G, Yang L, Ye N, et al. In-hospital acute kidney injury and atrial fibrillation: incidence, risk factors, and outcome. *Ren Fail*. Dec 2021;43(1):949-957. doi:10.1080/0886022X.2021.1939049

125. Katsogridakis E, Saha P, Diamantopoulos A, et al. Long-Term Effects of Acute Kidney Injury Following Endovascular Femoropopliteal Intervention: Insights From a Multicenter Trial. *J Endovasc Ther*. Aug 2024;31(4):634-642. doi:10.1177/15266028221136436

126. Yandrapalli S, Christy J, Malik A, et al. Impact of Acute and Chronic Kidney Disease on Heart Failure Hospitalizations After Acute Myocardial Infarction. *American Journal of Cardiology*. Feb 15 2022;165:1-11. doi:10.1016/j.amjcard.2021.10.041

127. Kutsal DA, Terzi S. Factors associated with acute kidney injury in patients undergoing transcatheter aortic valve implantation: Short-term outcomes and impact of right heart failure. *North Clin Istanb*. 2024;11(2):133-139. doi:10.14744/nci.2024.87864

128. Chou CL, Zheng CM, Chiu HW, et al. Impact of Acute Kidney Injury, Co-Existing with and without Chronic Kidney Disease on the Short-Term Adverse Outcomes Following Atherosclerotic Cardiovascular Disease Events in Patients with Diabetes. *J Multidiscip Healthc*. 2025;18:2019-2037. doi:10.2147/jmdh.S515884

129. Mutlu D, Ser OS, Strepkos D, et al. Acute Kidney Injury Requiring Dialysis Following Percutaneous Coronary Intervention: Insights From the PROGRESS-COMPLICATIONS Registry. *Catheter Cardiovasc Interv*. Sep 2025;106(3):1883-1891. doi:10.1002/ccd.70012

130. Schiffl H, Lang SM, Fischer R. Long-term outcomes of survivors of ICU acute kidney injury requiring renal replacement therapy: a 10-year prospective cohort study. *Clin Kidney J*. 2012-8 2012;5(4):297-302. doi:doi:10.1093/ckj/sfs070

131. Selby NM, Kolhe NV, McIntyre CW, et al. Defining the Cause of Death in Hospitalised Patients with Acute Kidney Injury. *Plos One*. 2012 2012;7(11)doi:doi:10.1371/journal.pone.0048580

132. Fernandes AR, Viegas MSR, Soares EQ, et al. Outcomes of acute kidney injury in a nephrology ward. *Int Urol Nephrol*. 2017-12 2017;49(12):2185-2193. doi:doi:10.1007/s11255-017-1716-6

133. Ohlmeier C, Schuchhardt J, Bauer C, et al. Risk of chronic kidney disease in patients with acute kidney injury following a major surgery: a US claims database analysis. *Clinical Kidney Journal*. Nov 30 2023;16(12):2461-2471. doi:10.1093/ckj/sfad148

134. Brar S, Ye F, James M, Hemmelgarn B, Klarenbach S, Pannu N. Statin Use and Survival After Acute Kidney Injury. *Kidney Int Rep*. 2016-11 2016;1(4):279-287. doi:doi:10.1016/j.ekir.2016.08.009

135. Balogun RA, Omotoso BA, Xin W, et al. Major Depression and Long-Term Outcomes of Acute Kidney Injury. *Nephron*. 2017 2017;135(1):23-30. doi:doi:10.1159/000449474

136. Lee BJ, Hsu CY, Parikh RV, et al. Non-recovery from dialysis-requiring acute kidney injury and short-term mortality and cardiovascular risk: a cohort study. *Bmc Nephrol*. Jun 11 2018;19doi:ARTN 134

10.1186/s12882-018-0924-3

137. Park S, Lee S, Kang MW, et al. Postdischarge Major Adverse Cardiovascular Events of ICU Survivors Who Received Acute Renal Replacement Therapy. *Crit Care Med*. Nov 2018;46(11):E1047-E1054. doi:10.1097/Ccm.0000000000003357

138. Rodriguez E, Arias-Cabrales C, Bermejo S, et al. Impact of Recurrent Acute Kidney Injury on Patient Outcomes. *Kidney Blood Press Res*. 2018;43(1):34-44. doi:10.1159/000486744

139. Lee S, Park S, Kang MW, et al. Postdischarge long-term cardiovascular outcomes of intensive care unit survivors who developed dialysis-requiring acute kidney injury after cardiac surgery. *Journal of Critical Care*. Apr 2019;50:92-98. doi:10.1016/j.jcrc.2018.11.028

140. Amatruda JG, Estrella MM, Garg AX, et al. Urine Alpha-1-Microglobulin Levels and Acute Kidney Injury, Mortality, and Cardiovascular Events following Cardiac Surgery. *American Journal of Nephrology*. Oct 2021;52(8):673-683. doi:10.1159/000518240

141. Vasquez-Rios G, Oh W, Lee S, et al. Molecular and clinical signatures in Acute Kidney Injury define distinct subphenotypes that associate with death, kidney, and cardiovascular events. *medRxiv*. 2021:2021.12.14.21267738. doi:10.1101/2021.12.14.21267738

142. Chen JJ, Lee TH, Kuo G, et al. Acute Kidney Disease After Acute Decompensated Heart Failure. *Kidney Int Rep*. Mar 2022;7(3):526-536. doi:10.1016/j.ekir.2021.12.033

143. Mansour SG, Bhatraju PK, Coca SG, et al. Angiopoietins as Prognostic Markers for Future Kidney Disease and Heart Failure Events after Acute Kidney Injury. *Journal of the American Society of Nephrology*. Mar 2022;33(3):613-627. doi:10.1681/Asn.2021060757

144. Cárdenas AC, Villanueva LS, Horrillo AS, Ramos PM, Ruano P, Quiroga B. Diastolic dysfunction is an independent predictor for cardiovascular events after an acute kidney injury. *Nefrologia*. Mar-Apr 2023;43(2):224-231. doi:10.1016/j.nefro.2021.11.001

145. Hirano Y, Fujikura T, Kono K, et al. Effect of rehabilitation on renal outcomes after acute kidney injury associated with cardiovascular disease: a retrospective analysis. *Bmc Nephrol*. Jul 12 2024;25(1)doi:ARTN 222

10.1186/s12882-024-03666-z

146. Jensen SK, Heide-Jorgensen U, Gammelager H, Birn H, Christiansen CF. Acute Kidney Injury Duration and 20-Year Risks of CKD and Cardiovascular Disease. *Kidney Int Rep*. Apr 2024;9(4):817-829. doi:10.1016/j.ekir.2024.01.034

147. Pan HC, Chen HY, Teng NC, et al. Recovery Dynamics and Prognosis After Dialysis for Acute Kidney Injury. *Jama Netw Open*. Mar 8 2024;7(3)doi:ARTN e240351

10.1001/jamanetworkopen.2024.0351

148. Wang D, Yan GL, Qiao Y, Sun RH. The relationship between perioperative serum albumin and contrast-induced acute kidney injury in patients after percutaneous coronary intervention. *Bmc Nephrol*. May 21 2024;25(1)doi:ARTN 173

10.1186/s12882-024-03608-9

149. Ahsan SI, Batool RM, Shahabi M, et al. Disparities and trends in cardiovascular disease mortality in patients with acute kidney injury in the United States from 1999-2020. *J Natl Med Assoc*. Aug 28 2025;doi:10.1016/j.jnma.2025.08.005

150. Adalbert S, Adelina M, Romulus T, et al. Acute kidney injury in peripheral arterial surgery patients: A cohort study. *Renal Failure*. 2013 2013;35(9):1236-1239. doi:doi:10.3109/0886022X.2013.823830

151. Andonovic M, Curle J, Traynor JP, et al. Impact of acute kidney injury on major adverse cardiovascular events in intensive care survivors. *BJA Open*. Dec 2023;8:100243. doi:10.1016/j.bjao.2023.100243

152. Andreis A, Budano C, Levis M, et al. Contrast-induced kidney injury: how does it affect long-term cardiac mortality? *Journal of Cardiovascular Medicine*. Nov 2017;18(11):908-915. doi:10.2459/Jcm.0000000000000543

153. Anzai A, Anzai T, Naito K, et al. Prognostic Significance of Acute Kidney Injury After Reperfused ST-Elevation Myocardial Infarction: Synergistic Acceleration of Renal Dysfunction and Left Ventricular Remodeling. *Journal of Cardiac Failure*. 2010 2010;16(5):381-389. doi:doi:10.1016/j.cardfail.2009.12.020

154. Armijo G, Estevez-Loureiro R, Carrasco-Chinchilla F, et al. Acute Kidney Injury After Percutaneous Edge-to-Edge Mitral Repair. *Journal of the American College of Cardiology*. Nov 24 2020;76(21):2463-2473. doi:10.1016/j.jacc.2020.09.582

155. Chalikias G, Serif L, Kikas P, et al. Long-term impact of acute kidney injury on prognosis in patients with acute myocardial infarction. *Int J Cardiol*. May 15 2019;283:48-54. doi:10.1016/j.ijcard.2019.01.070

156. Cho JS, Shim JK, Lee S, et al. Chronic progression of cardiac surgery associated acute kidney injury: Intermediary role of acute kidney disease. *J Thorac Cardiov Sur*. Feb 2021;161(2):681-+. doi:10.1016/j.jtcvs.2019.10.101

157. Florens N, Aymes E, Gauthier V, et al. Acute kidney injury as a key predictor of cardiovascular events in chronic kidney disease patients: the CKD-REIN study. *Clin Kidney J*. Dec 2024;17(12):sfae337. doi:10.1093/ckj/sfae337

158. Hansen MK, Gammelager H, Jacobsen CJ, et al. Acute Kidney Injury and Long-term Risk of Cardiovascular Events After Cardiac Surgery: A Population-Based Cohort Study. *J Cardiothor Vasc An*. Jun 2015;29(3):617-625. doi:10.1053/j.jvca.2014.08.020

159. Heitmann LA, Sveinsdottir N, Helgadottir S, Indridason OS, Gudbjartsson T, Helgason D. The Role of Other Major Postoperative Complications in Determining Outcomes of Acute Kidney Injury After Coronary Artery Bypass Surgery. *Acta Anaesthesiologica Scandinavica*. 2025;69(8):e70110. doi:<https://doi.org/10.1111/aas.70110>

160. Kimura T, Obi Y, Yasuda K, et al. Effects of chronic kidney disease and post-angiographic acute kidney injury on long-term prognosis after coronary artery angiography. *Nephrology Dialysis Transplantation*. Jun 2011;26(6):1838-1846. doi:10.1093/ndt/gfq631

161. Ko T, Higashitani M, Sato A, et al. Impact of Acute Kidney Injury on Early to Long-Term Outcomes in Patients Who Underwent Surgery for Type A Acute Aortic Dissection. *American Journal of Cardiology*. Aug 1 2015;116(3):463-468. doi:10.1016/j.amjcard.2015.04.043

162. Korczak A, Morawiec R, Stegienta M, et al. Acute kidney injury as the most important predictor of poor prognosis after interventional treatment for aortic stenosis. *Kardiol Pol*. Aug 3 2022;80(10):1032-1038. doi:10.33963/KP.a2022.0182

163. Lee J, Liu JJ, Liu S, et al. Acute kidney injury predicts the risk of adverse cardio renal events and all cause death in southeast Asian people with type 2 diabetes. *Sci Rep*. Nov 7 2024;14(1):27027. doi:10.1038/s41598-024-77981-8

164. Leistner DM, Münch C, Steiner J, et al. Impact of acute kidney injury in elderly (≥80 years) patients undergoing percutaneous coronary intervention. *J Interv Cardiol*. Dec 2018;31(6):792-798. doi:10.1111/joic.12547

165. Lentini P, Zanoli L, Fatuzzo P, et al. Stroke volume variation and serum creatinine changes during abdominal aortic aneurysm surgery: a time-integrated analysis. *Journal of Nephrology*. Aug 2018;31(4):561-569. doi:10.1007/s40620-018-0467-5

166. Lunyera J, Clare RM, Chiswell K, et al. Association of Acute Kidney Injury and Cardiovascular Disease Following Percutaneous Coronary Intervention: Assessment of Interactions by Race, Diabetes, and Kidney Function. *American Journal of Kidney Diseases*. Jun 2023;81(6):707-716. doi:10.1053/j.ajkd.2022.12.013

167. Monseu M, Gand E, Saulnier PJ, et al. Acute kidney injury predicts major adverse outcomes in diabetes: Synergic impact with low glomerular filtration rate and albuminuria. *Diabetes Care*. 2015 2015;38(12):2333-2340. doi:doi:10.2337/dc15-1222

168. Nagaraja V, Rihal CS, Reeder G, Nath KA, Lewis BR, Singh M. Impact of Renal Function and Acute Kidney Injury on Long-term Outcomes After Percutaneous Coronary Intervention. *Mayo Clin Proc*. Aug 2025;100(8):1309-1318. doi:10.1016/j.mayocp.2025.01.020

169. Nakamura T, Watanabe M, Sugiura J, et al. Prognostic impact and predictors of persistent renal dysfunction in acute kidney injury after percutaneous coronary intervention for acute myocardial infarction. *Sci Rep-Uk*. Mar 15 2024;14(1)doi:ARTN 6299

10.1038/s41598-024-56929-y

170. Ng AK, Ng PY, Ip A, et al. Impact of contrast-induced acute kidney injury on long-term major adverse cardiovascular events and kidney function after percutaneous coronary intervention: insights from a territory-wide cohort study in Hong Kong. *Clin Kidney J*. Feb 2022;15(2):338-346. doi:10.1093/ckj/sfab212

171. Ozaki Y, Nakao K, Kimura K, et al. Influence of acute kidney injury and chronic kidney disease on 3-year clinical outcomes in patients with AMI treated by contemporary PCI and optimal medical therapy; insights from J-MINUET study. *European Heart Journal*. 2024;45(Supplement_1)doi:10.1093/eurheartj/ehae666.2421

172. Parikh CR, Puthumana J, Shlipak MG, et al. Relationship of kidney injury biomarkers with long-term cardiovascular outcomes after cardiac surgery. *Journal of the American Society of Nephrology*. 2017 2017;28(12):3699-3707. doi:doi:10.1681/ASN.2017010055

173. Ruzzarin A, Muraglia S, Fabris E, et al. Impact of Contrast-Associated Acute Kidney Injury on One-Year Outcomes in Very Elderly STEMI Patients: Insights From a Multicenter Registry in Northern Italy. *Angiology*. Feb 20 2024:33197241233771. doi:10.1177/00033197241233771

174. Saratzis A, Harrison S, Barratt J, Sayers RD, Sarafidis PA, Bown MJ. Intervention Associated Acute Kidney Injury and Long-Term Cardiovascular Outcomes. *American Journal of Nephrology*. 2015 2015;42(4):285-294. doi:doi:10.1159/000440986

175. Suzuki T, Kohsaka S, Spertus JA, et al. Risk-Stratified Prognostic Implications of Contrast-Associated Acute Kidney Injury After Percutaneous Coronary Intervention. *Jacc-Adv*. Jul 2025;4(7)doi:ARTN 101899

10.1016/j.jacadv.2025.101899

176. Tajti P, Ayoub M, Ahres A, et al. Procedural outcomes of chronic total occlusion percutaneous coronary interventions in patients with acute kidney injury. *Cardiol J*. Jan 2024;31(1):84-94. doi:10.5603/CJ.a2022.0121

177. Tsagalis G, Akrivos T, Alevizaki M, et al. Long-term prognosis of acute kidney injury after first acute stroke. *Clinical Journal of the American Society of Nephrology*. 2009 2009;4(3):616-622. doi:doi:10.2215/CJN.04110808

178. Valle JA, McCoy LA, Maddox TM, et al. Longitudinal Risk of Adverse Events in Patients With Acute Kidney Injury After Percutaneous Coronary Intervention: Insights From the National Cardiovascular Data Registry. *Circ Cardiovasc Interv*. 2017-4 2017;10(4)doi:doi:10.1161/circinterventions.116.004439

179. Wu MZ, Chen Y, Au WK, et al. Predictive value of acute kidney injury for major adverse cardiovascular events following tricuspid annuloplasty: A comparison of three consensus criteria. *J Cardiol*. Sep-Oct 2018;72(3-4):247-254. doi:10.1016/j.jjcc.2018.01.018

180. Zhang L, Xu JR, Li XY, et al. Risk Factors and Outcomes of AKI after LAAC Operation: A Single-Center Observational Study from Mainland China. *Rev Cardiovasc Med*. Sep 9 2022;23(9)doi:ARTN 306

10.31083/j.rcm2309306

181. Chen Z, Mao Q, Xiang L, et al. Iodixanol-associated acute kidney injury and prognosis in patients undergoing elective percutaneous coronary intervention: a prospective, multi-center study. *Eur Radiol*. Dec 2023;33(12):9444-9454. doi:10.1007/s00330-023-09964-8

182. Hansen MK, Gammelager H, Mikkelsen MM, et al. Post-operative acute kidney injury and five-year risk of death, myocardial infarction, and stroke among elective cardiac surgical patients: a cohort study. *Critical Care*. 2013;17(6)doi:ARTN R292

10.1186/cc13158

183. Rydén L, Ahnve S, Bell M, et al. Acute kidney injury after coronary artery bypass grafting and long-term risk of myocardial infarction and death. *International Journal of Cardiology*. 2014 2014;172(1):190-195. doi:doi:10.1016/j.ijcard.2014.01.013

184. Huepenbecker SP, Iniesta MD, Munsell MF, et al. No increase in chronic kidney disease after postoperative acute kidney injury in gynecologic oncologic ERAS surgery. *Gynecol Oncol*. Jul 2025;198:168-175. doi:10.1016/j.ygyno.2025.06.002

185. Yan GY, Lou JF, Liu X, Yuan H, Lu Y. Cardio- and Cerebrovascular Outcomes of Postoperative Acute Kidney Injury in Noncardiac Surgical Patients With Hypertension. *Front Pharmacol*. Aug 27 2021;12doi:ARTN 696456

10.3389/fphar.2021.696456

186. Gammelager H, Christiansen CF, Johansen MB, Tønnesen E, Jespersen B, Sørensen HT. Three-year risk of cardiovascular disease among intensive care patients with acute kidney injury: A population-based cohort study. *Critical Care*. 2014 2014;18(5)doi:doi:10.1186/s13054-014-0492-2

187. Go AS, Hsu CY, Yang JR, et al. Acute Kidney Injury and Risk of Heart Failure and Atherosclerotic Events. *Clinical Journal of the American Society of Nephrology*. Jun 7 2018;13(6):833-841. doi:10.2215/Cjn.12591117

188. Olsson D, Sartipy U, Braunschweig F, Holzmann MJ. Acute kidney injury following coronary artery bypass surgery and long-term risk of heart failure. *Circulation: Heart Failure*. 2013 2013;6(1):83-90. doi:doi:10.1161/CIRCHEARTFAILURE.112.971705

189. Horne KL, Viramontes-Hröner D, Packington R, et al. A comprehensive description of kidney disease progression after acute kidney injury from a prospective, parallel-group cohort study. *Kidney International*. Dec 2023;104(6):1185-1193. doi:10.1016/j.kint.2023.08.005

190. Ikizler TA, Parikh CR, Himmelfarb J, et al. A prospective cohort study of acute kidney injury and kidney outcomes, cardiovascular events, and death. *Kidney International*. Feb 2021;99(2):456-465. doi:10.1016/j.kint.2020.06.032

191. Brar S, Liu KD, Go AS, et al. Prospective Cohort Study of Renin-Angiotensin System Blocker Usage after Hospitalized Acute Kidney Injury. *Clinical Journal of the American Society of Nephrology*. Jan 7 2021;16(1):26-36. doi:10.2215/Cjn.10840720

192. MacLaughlin HL, Pike M, Selby NM, et al. Body mass index and chronic kidney disease outcomes after acute kidney injury: a prospective matched cohort study. *Bmc Nephrol*. May 28 2021;22(1)doi:ARTN 200

10.1186/s12882-021-02400-3

193. Li Q, Shi SS, Lu XZ, et al. Effect of kidney disease on all-cause and cardiovascular mortality in patients undergoing coronary angiography. *Renal Failure*. Dec 31 2023;45(1)doi:Artn 2195950

10.1080/0886022x.2023.2195950

194. Mezhonov EM, Vialkina IA, Vakulchik KA, Shalaev SV. Acute kidney injury in patients with ST-segment elevation acute myocardial infarction: Predictors and outcomes. *Saudi J Kidney Dis T*. Mar-Apr 2021;32(2):318-327. doi:Pmid 35017324

10.4103/1319-2442.335442

195. Huber M, Ozrazgat-Baslanti T, Thottakkara P, Scali S, Bihorac A, Hobson C. Cardiovascular-specific mortality and kidney disease in patients undergoing vascular surgery. *JAMA Surgery*. 2016 2016;151(5):441-450. doi:doi:10.1001/jamasurg.2015.4526

196. Lysak N, Hashemighouchani H, Davoudi A, et al. Cardiovascular death and progression to end-stage renal disease after major surgery in elderly patients. *Bjs Open*. Feb 2020;4(1):145-156. doi:10.1002/bjs5.50232

197. Ozrazgat-Baslanti T, Thottakkara P, Huber M, et al. Acute and Chronic Kidney Disease and Cardiovascular Mortality After Major Surgery. *Ann Surg*. 2016-12 2016;264(6):987-996. doi:doi:10.1097/sla.0000000000001582

198. Niittyvuopio M, Vaara S, Ohtonen P, Pettilä V, Liisanantti J, Ala-Kokko T. Causes of death for intensive care survivors with and without acute kidney injury in 5-year follow-up. *Acta Anaesthesiologica Scandinavica*. Apr 2021;65(4):507-514. doi:10.1111/aas.13754
